# Proteogenomic analysis of Inhibitor of Differentiation 4 (ID4) in basal-like breast cancer

**DOI:** 10.1186/s13058-020-01306-6

**Published:** 2020-06-11

**Authors:** Laura A. Baker, Holly Holliday, Daniel Roden, Christoph Krisp, Sunny Z. Wu, Simon Junankar, Aurelien A. Serandour, Hisham Mohammed, Radhika Nair, Geetha Sankaranarayanan, Andrew M. K. Law, Andrea McFarland, Peter T. Simpson, Sunil Lakhani, Eoin Dodson, Christina Selinger, Lyndal Anderson, Goli Samimi, Neville F. Hacker, Elgene Lim, Christopher J. Ormandy, Matthew J. Naylor, Kaylene Simpson, Iva Nikolic, Sandra O’Toole, Warren Kaplan, Mark J. Cowley, Jason S. Carroll, Mark Molloy, Alexander Swarbrick

**Affiliations:** 1 The Kinghorn Cancer Centre and Cancer Research Division, Garvan Institute of Medical Research, Darlinghurst, NSW 2010 Australia; 2 St Vincent’s Clinical School, Faculty of Medicine, UNSW Sydney, Sydney, NSW 2052 Australia; 3 Australian Proteome Analysis Facility (APAF), Department of Chemistry and Biomolecular Sciences, Macquarie University, Sydney, Australia; 4 Mass Spectrometric Proteome Analysis, Institute of Clinical Chemistry and Laboratory Medicine, University Medical Center Hamburg-Eppendorf, 20246 Hamburg, Germany; 5 Cancer Research UK, The University of Cambridge, Li Ka Shing Centre, Robinson Way, Cambridge, CB2 0RE UK; 6 Rajiv Gandhi Centre for Biotechnology, Thycaud Post, Poojappura, Thiruvananthapuram, Kerala 695014 India; 7 Centre for Clinical Research, Faculty of Medicine, The University of Queensland, Brisbane, QLD Australia; 8 Pathology Queensland, The Royal Brisbane and Women’s Hospital, Herston, , Brisbane, QLD Australia; 9 Department of Tissue Pathology and Diagnostic Oncology, Royal Prince Alfred Hospital, Camperdown, NSW 2050 Australia; 10 Sydney Medical School, University of Sydney, Sydney, NSW 2006 Australia; 11 National Cancer Institute, National Institutes of Health, 9609 Medical Center Drive, Bethesda, MD 20892 USA; 12 School of Women’s and Children’s Health, University of New South Wales, and Gynaecological Cancer Centre, Royal Hospital for Women, Sydney, NSW Australia; 13 School of Medical Sciences and Bosch Institute, Sydney Medical School, The University of Sydney, Sydney, NSW 2006 Australia; 14 Victorian Centre for Functional Genomics, Peter MacCallum Cancer Centre, Melbourne, VIC 3000 Australia; 15 Sir Peter MacCallum Department of Oncology, University of Melbourne, Melbourne, VIC 3052 Australia

**Keywords:** Breast cancer, Transcription factor, Helix-loop-helix, DNA damage

## Abstract

**Background:**

Basal-like breast cancer (BLBC) is a poorly characterised, heterogeneous disease. Patients are diagnosed with aggressive, high-grade tumours and often relapse with chemotherapy resistance. Detailed understanding of the molecular underpinnings of this disease is essential to the development of personalised therapeutic strategies. Inhibitor of differentiation 4 (ID4) is a helix-loop-helix transcriptional regulator required for mammary gland development. ID4 is overexpressed in a subset of BLBC patients, associating with a stem-like poor prognosis phenotype, and is necessary for the growth of cell line models of BLBC through unknown mechanisms.

**Methods:**

Here, we have defined unique molecular insights into the function of ID4 in BLBC and the related disease high-grade serous ovarian cancer (HGSOC), by combining RIME proteomic analysis, ChIP-seq mapping of genomic binding sites and RNA-seq.

**Results:**

These studies reveal novel interactions with DNA damage response proteins, in particular, mediator of DNA damage checkpoint protein 1 (MDC1). Through MDC1, ID4 interacts with other DNA repair proteins (γH2AX and BRCA1) at fragile chromatin sites. ID4 does not affect transcription at these sites, instead binding to chromatin following DNA damage. Analysis of clinical samples demonstrates that ID4 is amplified and overexpressed at a higher frequency in *BRCA1*-mutant BLBC compared with sporadic BLBC, providing genetic evidence for an interaction between ID4 and DNA damage repair deficiency.

**Conclusions:**

These data link the interactions of ID4 with MDC1 to DNA damage repair in the aetiology of BLBC and HGSOC.

## Background

Breast cancer is a heterogeneous disease. Gene expression signatures delineate five major subtypes with distinct pathologies, treatment strategies and clinical outcomes [1, 2]. The basal-like subtype (BLBC), accounting for ~ 18% of diagnoses, is a subtype with particularly poor prognosis, largely due to the molecular and clinical heterogeneity of these tumours, and the corresponding lack of targeted therapeutics. The molecular drivers of BLBC are poorly understood, thus there are limited available targeted therapies.

One such molecular driver of a subset of BLBCs is mutation in the breast and ovarian cancer susceptibility gene (*BRCA1*) [3, 4]. Mutations in *BRCA1* occur in approximately 0.25% of European women, predisposing them to breast and ovarian cancer, particularly to poor prognosis subtypes including BLBC and high-grade serous ovarian cancer (HGSOC) [3]. These two subtypes of cancer are similar in terms of their gene expression profiles and genetic dependencies, and thus present similar sensitivity to therapeutic targeting [5–7]. BRCA1 has many cellular functions including transcription and gene splicing, yet is best known for its role in mediating DNA damage repair [8, 9]. BRCA1 coordinates efficient repair of double stranded DNA breaks through the homologous recombination (HR) pathway [9]. In the absence of functional BRCA1, cells accumulate mutations and genomic instability and demonstrate an increased frequency of genomic rearrangements [10].

Another molecular driver of BLBC is the Helix-Loop-Helix (HLH) transcriptional regulator inhibitor of differentiation 4 (ID4). We and others have previously shown ID4 to be important for both mammary gland development and also for the aetiology of BLBC [11–13]. ID4 is overexpressed in a subset of BLBC patients, marking patients with poor survival outcome, and is necessary for the growth of BLBC cell lines [11, 14–21]. Precisely how ID4 mediates this function in BLBC is unclear.

ID proteins (ID1–4) lack a basic DNA binding domain, and thus, their classical mechanism of action is believed to entail dominant-negative regulation of canonical binding partners, basic HLH (bHLH) transcription factors. ID proteins dimerise with bHLH proteins and prevent them from interacting with DNA, affecting the transcription of lineage-specific genes [22–26]. Yet this model of ID protein function is largely based on evidence from studies of ID1–3 in non-transformed fibroblasts, neural and embryonic tissue. ID proteins are tissue specific in their expression and function, and hence, this model may not apply to all four ID proteins across various tissues and in disease [25, 27–32]. Indeed, contrary mechanisms have been described for ID2 in liver regeneration, with ID2 interacting with chromatin at the c-Myc promoter as part of a multi-protein complex to repress c-Myc gene expression [33, 34], and ID4 has been shown to bind to and suppress activity of the ERα promoter and regions upstream of the ERα and FOXA1 genes in mouse mammary epithelial cells [12]. These data suggest that despite lacking a DNA binding domain, ID proteins may interact with chromatin complexes under certain conditions. However, no studies have systematically mapped the protein or chromatin interactomes for any ID family member.

To this end, we applied chromatin immunoprecipitation-sequencing (ChIP-seq) to interrogate the ID4-chromatin binding sites and rapid immunoprecipitation and mass spectrometry of endogenous proteins (RIME) to determine the ID4 protein interactome. In addition, ID4 knockdown and RNA-sequencing analysis was used to determine transcriptional targets of ID4. These analyses reveal a novel link between ID4 with the DNA damage repair apparatus.

## Methods

### Mammalian cell culture growth conditions

Cell lines were obtained from American Type Culture Collection and verified using cell line fingerprinting. HCC70 and HCC1937 cell lines were cultured in RPMI 1640 (Thermo Fisher Scientific) supplemented with 10% (v/v) FBS, 20 mM HEPES (Thermo Fisher Scientific) and 1 mM Sodium Pyruvate (Thermo Fisher Scientific). MDA-MB-468 and OVKATE cell lines were cultured in RPMI 1640 (Thermo Fisher Scientific) supplemented with 10% (v/v) FBS, 20 mM HEPES (Thermo Fisher Scientific) and 0.25% (v/v) insulin (Human) (Clifford Hallam Healthcare). Cells stably transduced with SMARTChoice lentiviral vectors were grown in the presence of 1 μg/mL puromycin.

### Imaging

Immunohistochemistry images were obtained using an inverted epifluorescence microscope (Carl Zeiss, ICM-405, Oberkochem, Germany). Images were captured by the Leica DFC280 digital camera system (Leica Microsystems, Wetzlar, Germany). The Leica DM 5500 Microscope with monochrome camera (DFC310Fx) or Leica DMI SP8 Confocal with 4 lasers (405 nm, 488 nm, 552 nm and 638 nm) and two PMT detectors were used to capture standard fluorescent and confocal images.

### SMARTChoice inducible lentiviral system

ID4 and control lentiviral shRNA constructs (SMARTchoice) were purchased commercially (Dharmacon, GE, Lafayette, CO, USA). Successfully transfected cells were selected using puromycin resistance (constitutive under the humanEF1a promoter). For ID4 knockdown analysis, HCC70 cells with SMARTChoice shID4 #1 #178657 (VSH6380-220912204), SMARTChoice shID4 #2 #703009 (VSH6380-221436556), SMARTChoice shID4 #3 #703033 (VSH6380-221436580), SMARTChoice shNon-targeting (VSC6572) and mock-infected cells were used. Cells were treated with vehicle control or with 1 μg/mL doxycycline for 72 h before harvesting protein and RNA directly from adherent cells. The SMARTChoice shID4 #2 #703009 (VSH6380-221436556) produced the highest level of ID4 knockdown and was used for further analysis.

### Non-lethal DNA damage induction with ionising radiation

Cells were seeded at 2.5 × 10^5^ (HCC70) or 2.2 × 10^5^ (MDA-MB-468) cells/well in a 6-well plate in normal growth medium. One day post seeding, cells were exposed to 2–5 Gy of ionising radiation using an X-RAD 320 Series Biological Irradiator (Precision X-Ray, CT, USA). Cells were returned to normal tissue culture incubation conditions and harvested at designated time points.

### Gene expression analysis

Total RNA was prepared for using the miRNeasy RNA extraction kit (Qiagen), according to the manufacturer’s instructions. cDNA was generated from 1000 ng RNA using the Transcriptor First Strand cDNA Synthesis Kit (Roche) using oligo-dT primers and following the manufacturer’s instructions. qPCR analysis was used to analyse mRNA expression levels using Taqman probes (Applied Biosystems/Life Technologies) as per the manufacturer’s specifications (Table 1) using an ABI PRISM 7900 HT machine. qPCR data was analysed using the ΔΔCt method [35].

**Table 1:** Genes analysed and the corresponding Taqman assay used to analyse their expression level

Gene	Taqman assay
ID4	Hs02912975_g1
B2M	Hs99999907_m1
GAPDH	Hs02758991_s1
NEAT1	Hs01008264_s1
MALAT1	Hs00273907_s1
ELF3	HS00963881_M1
GBA	HS00986836_G1
ZFP36L1	Hs00245183_m1
FAIM	HS00216756_M1

### Protein analysis

Cells were lysed, unless specified, using RIPA [0.88% (w/v) Sodium Chloride, 1% (v/v) Triton X-100, 0.5% (w/v) Sodium Deoxycholate, 0.1% (w/v) SDS, 0.61% (v/v) Tris (Hydroxymethyl) Aminomethane and protease and phosphatase inhibitors (Roche)] or Lysis Buffer 5 (10 mM Tris pH 7.4, 1 mM EDTA, 150 mM NaCl, 1% Triton X-100 and protease and phosphatase inhibitors). If required, protein was quantified using the Pierce BCA Protein Assay Kit (Thermo Fisher Scientific) according to the manufacturer’s instructions. Western blotting analysis was conducted as previously described [11]. MDC1 protein was analysed using 3–8% tris/acetate gels and PVDF nitrocellulose membrane for MDC1 analysis (BioRad). All other proteins were analysed using the LiCor Odyssey system (Millenium Science, Mulgrave, VIC, Australia). Protein expression was analysed using antibodies targeting ID4 (Biocheck anti-ID4 rabbit monoclonal BCH-9/82-12, 1:40,000), β-Actin (Sigma anti-Actin mouse monoclonal A5441, 1:5000) and MDC1 (Sigma anti-MDC1 mouse monoclonal M2444, 1:1000).

### Co-immunoprecipitation

Co-immunoprecipitations (IP) were conducted using 10 μL per IP Pierce Protein A/G magnetic beads (Thermo Fisher Scientific) with 2 μg of antibody: IgG rabbit polyclonal (Santa Cruz sc-2027), ID4 (1:1 mix of rabbit polyclonal antibodies: Santa Cruz L-20: sc-491 and Santa Cruz H-70: sc-13047) and MDC1 (rabbit polyclonal antibody Merck Millipore #ABC155). Beads and antibody were incubated at 4 °C on a rotating platform for a minimum of 4 h. Beads were then washed three times in lysis buffer before cell lysate was added to the tube. Lysates were incubated with beads overnight at 4 °C on a rotating platform. Beads were washed three times in lysis buffer and resuspended in 2× NuPage sample reducing buffer (Life Technologies) and 2× NuPage sample running buffer (Life Technologies) and heated to 85 °C for 10 min. Beads were separated on a magnetic rack, and supernatant was analysed by western blotting as described above.

### Rapid immunoprecipitation and mass spectrometry of endogenous proteins (RIME)

Cells were fixed using paraformaldehyde (PFA) (ProSciTech, Townsville, QLD, Australia) and prepared for RIME [36] and ChIP-seq [37, 38] as previously described. Cross-linking was performed for 7 min for RIME experiments and 10 min for ChIP-seq, ChIP-exo and ChIP-qPCR experiments. Samples were sonicated using a Bioruptor Standard (Diagenode, Denville, NJ, USA) for 30–35 cycles of 30 s on/30 s off (sonication equipment kindly provided by Prof. Merlin Crossley, UNSW). IP was conducted on 60 (ChIP-seq/ ChIP-exo) to 120 (RIME) million cells using 100 μL beads/20 μg antibody. Correct DNA fragment size of 100–500 bp was determined using 2% agarose gel electrophoresis.

Patient-derived xenograft tumour models were cross-linked at 4 °C for 20 min in a solution of 1% Formaldehyde (ProSciTech), 50 mM Hepes–KOH, 100 mM NaCl, 1 mM EDTA, 0.5 mM EGTA and protease inhibitors (H. Mohammed, personal communication). Samples (0.5 mg of starting tumour weight) were dissociated using a Polytron PT 1200E tissue homogeniser (VWR) and sonicated using the Branson Digital Sonifer probe sonicator (Branson Ultrasonics, Danbury, CT, USA) with a microtip attachment for 3–4 cycles of 10 × [0.1 s on, 0.9 s off].

*Mass spectrometry analysis* was conducted at the Australian Proteomic Analysis Facility (APAF) at Macquarie University (NSW, Australia) [39]. Briefly, samples were denatured in 100 mM triethylammonium bicarbonate and 1% w/v sodium deoxycholate; disulfide bonds were reduced in 10 mM dithiotreitol and alkylated in 20 mM iodo acetamide, and proteins digested on the dynabeads using trypsin. After C18 reversed phase (RP) StageTip sample clean up, peptides were submitted to nano liquid chromatography coupled mass spectrometry (MS) (nanoLC-MS/MS) characterisation. MS was performed using a TripleToF 6600 (SCIEX, MA, USA) coupled to a nanoLC Ultra 2D HPLC with cHipLC system (SCIEX). Peptides were separated using a 15-cm chip column (ChromXP C18, 3 μm, 120 Å) (SCIEX). The mass spectrometer was operated in positive ion mode using a data-dependent acquisition method (DDA) and data-independent acquisition mode (DIA or SWATH) both using a 60-min acetonitrile gradient from 5 to 35%. DDA was performed of the top 20 most intense precursors with charge stages from 2+ to 4+ with a dynamic exclusion of 30 s. SWATH-MS was acquired using 100 variable precursor windows based on the precursor density distribution in data-dependent mode. MS data files were processed using ProteinPilot v.5.0 (SCIEX) to generate mascot generic files. Processed files were searched against the reviewed human SwissProt reference database using the Mascot (Matrix Science, MA, USA) search engine version 2.4.0. Searches were conducted with tryptic specificity, carbamidomethylation of cysteine residues as static modification and the oxidation of methionine residues as a dynamic modification. Using a reversed decoy database, false discovery rate was set to less than 1% and above the Mascot-specific peptide identity threshold. For SWATH-MS processing, ProteinPilot search outputs from DDA runs were used to generate a spectral library for targeted information extraction from SWATH-MS data files using PeakView v2.1 with SWATH MicroApp v2.0 (SCIEX). Protein areas, summed chromatographic area under the curve of peptides with extraction FDR ≤ 1%, were calculated and used to compare protein abundances between bait and control IPs.

### Chromatin immunoprecipitation-quantitative real-time PCR analysis

Chromatin immunoprecipitation (ChIP) was conducted as described previously [37]; however, following overnight IP, the samples were processed using a previously described protocol [40].

DNA was purified then quantified using quantitative real-time PCR analysis. Control regions analysed and primers used are listed in Table S4.

Relative enrichment of each region/primer set was calculated by taking an average of each duplicate reaction. The input Ct value was subtracted from the sample Ct value and the Ct converted using the respective PE for each primer set. The relative ChIP enrichment is then calculated by dividing the gene region of interest by the specific control region that is negative for both ID4 and H3K4Me3 binding (IFF01/NOP2 #1 primer). The formula for this normalisation is below:

$$ \Phi \mathrm{Ct}={\mathrm{Ct}}_{\mathrm{region}\ \mathrm{of}\ \mathrm{interest}}-{\mathrm{Ct}}_{\mathrm{input}\ \mathrm{region}} $$

$$ \mathrm{ChIP}\ \mathrm{enrichment}={\mathrm{PE}}^{\left[-\Phi \mathrm{Ct}\left(\mathrm{region}\ \mathrm{of}\ \mathrm{interest}\right)\right]}-{\mathrm{PE}}^{\left[-\Phi \mathrm{Ct}\left(\mathrm{IFF}01\right)\right]} $$

A sample was considered to be enriched if the fold-change over IgG control for each region was > 2.

### Chromatin immunoprecipitation-sequencing

*Chromatin immunoprecipitation and sequencing* (ChIP-seq) was conducted as previously described [37]. Samples were prepared and sequenced at Cancer Research United Kingdom (CRUK), Cambridge, UK. Antibody conditions for ChIP are the same as those used for RIME, with the addition of antibodies targeting H3K4Me3 (Active Motif #39159) and γH2AX (Ser139) (1:1 mix of Cell Signalling #2577 and Merck Millipore clone JBW301). Samples were sequenced at CRUK using an Illumina HiSeq 2500 single-end 50-bp sequencing. Quality control was conducted using FastQC [41] and sequencing adapters trimmed using cutadapt [42]. Reads were aligned using Bowtie for Illumina v0.12.7 [43] followed by Sam-to-Bam conversion tool [44] and alignment using Bwa v0.705a [44]. Alignment statistics were generated using samtools flagstat [version 0.1.18 (r982:295)] [44]. ChIP-seq peaks were called using the peak calling algorithm HOMER v4.0 and MACS v1.4.2 [45, 46].

*Chromatin immunoprecipitation-exonuclease sequencing* (ChIP-exo) was conducted as previously described [38]. Samples were prepared and sequenced at CRUK.

### qPCR analysis of ChIP DNA

Publicly available H4K3Me3 ChIP-sequencing data and the ID4 ChIP-sequencing data generated in this project were visualised using UCSC Genome Browser (genome.ucsc.edu and [47]). Regions of positive and negative enrichment were selected and the 500–1000-bp DNA sequence was imported into Primer3, a primer design interface, web version 4.0.0 [48]. Primers were designed with a minimum primer amplicon length of 70 bp. Primers were confirmed to align with specific DNA segments by conducting an in silico PCR using UCSC Genome Browser (genome.ucsc.edu and [47]). Oligo primers were ordered from Integrated DNA Technologies (Singapore). Primers were tested to determine adequate primer efficiency (between 1.7 and 2.3). All assays were set up using an EPmotion 5070 robot (Eppendorf, AG, Germany) and run on an ABI PRISM 7900 HT machine (Life Technologies, Scoresby, VIC, Australia). Briefly, reactions were performed in triplicate in a 384-well plate. Each reaction consisted of 1 μL 5 μM Forward primer, 1 μL 5 μM Reverse primer, 5 μL SYBR Green PCR Mastermix (Thermo Fisher) and 3 μL DNA. A standard curve was created using unsonicated, purified DNA extracted from the HCC70 cell line in 10-fold dilutions (1, 0.1, 0.01, 0.001, 0.0001).

PCR cycling was as follows: 1 cycle at 50 °C for 2 min, 1 cycle at 95 °C for 10 min, followed by 40 cycles of 95 °C for 15 s and 60 °C for 1 min. A dissociation step was conducted at 95 °C for 15 s and 60 °C for 15 s. Data was analysed and a standard curve created using SDS 2.3 software (Applied Biosystems). The slope was used to calculate the PE using the qPCR Primer Efficiency Calculator (Thermo Fisher Scientific, available at thermofisher.com).

### Patient-derived xenograft and histology

All experiments involving mice were performed in accordance with the regulations of the Garvan Institute Animal Ethics Committee. NOD.CB17-Prkdc^scid^/Arc mice were sourced from the Australian BioResources Ltd. (Moss Vale, NSW, Australia). Assoc. Prof Alana Welm (Oklahoma Medical Research Foundation) kindly donated the patient-derived xenografts (PDX) models used in this study. Models were maintained as described elsewhere [49]. Tumour chunks were transplanted into the 4th mammary gland of 5-week-old recipient NOD.CB17-Prkdc^scid^/Arc mice. Tumours were harvested at ethical endpoint, defined as having a tumour approximately 1 mm^3^ in size or deterioration of the body condition score. At harvest, a cross-section sample of the tumour was fixed in 10% neutral buffered formalin (Australian Biostain, Traralgon, VIC, Australia) overnight before transfer to 70% ethanol for storage at 4 °C before histopathological analysis. The formalin fixed paraffin embedded (FFPE) blocks were cut in 4-μm-thick sections and stained for ID4 (Biocheck BCH-9/82-12, 1:1000 for 60 min following antigen retrieval using pressure cooker 1699 for 1 min, Envision Rabbit secondary for 30 min). Protein expression was scored by a pathologist using the H-score method [50].

### Fluorescent in situ hybridisation

Tissue sections were analysed using fluorescent in situ hybridisation (FISH) to examine the genomic region encoding *ID4* (6p22.3). *ID4* FISH Probe (Orange 552–576 nm, Empire Genomics, NY, USA) was compared to the control probe *CEP6* (Chromosome 6, Green 5-Fluorescein dUTP). This *CEP6* probe marks a control region on the same chromosome as *ID4* and is used to normalise *ID4* copy number. Breast pathologist Dr. Sandra O’Toole oversaw the FISH quantification for all samples.

### Immunofluorescence and proximity ligation assays

#### Immunofluorescence

Cells were seeded on glass coverslips (Coverglass, 13 mm, VITLAB, Germany). At harvest, media was removed, and cells were washed twice with PBS without salts and fixed in 4% paraformaldehyde (PFA) (ProSciTech) for 10 min. Cells were again washed twice with PBS without salts (Thermo Fisher Scientific) before permeabilising for 15 min with 1% Triton-X (Sigma-Aldrich) in PBS and then blocking with 5% BSA in PBS without salts for 1 h at room temperature. Cells were washed twice with PBS without salts, and antibodies were applied overnight at 4 °C: ID4 (Biocheck BCH-9/82-12, 1:1000), MDC1 (Sigma-Aldrich M2444, 1:1000), BRCA1 (Merck Millipore (Ab-1), MS110, 1:250), γH2AX (Ser139) (Merck Millipore clone JBW301 05-636, 1:300), FLAG (Sigma-Aldrich M2, 1:500) and V5 (Santa Cruz sc-58052, 1:500). Cells were washed twice with PBS without salts; then, secondary antibodies were applied for 1 h at room temperature. Cells were washed twice with PBS without salts, with the second wash containing DAPI (1:500 dilution) and phalloidin (1:1000 dilution) (CytoPainter Phalloidin-iFluor 633 Reagent Abcam ab176758). Cells were then mounted on slides using 4 μL of Prolong Diamond (Thermo Fisher Scientific).

#### Duolink proximity ligation assay analysis (PLA)

PLA was conducted using Duolink PLA technology with Orange mouse/rabbit probes (Sigma-Aldrich, DUO92102) according to the manufacturer’s instructions. Images were captured using SP8 6000 confocal imaging with 0.4um Z-stacks. Maximum projects were made for each image (100–200 cells) and quantified using FIJI by ImageJ [51] as described previously [52]. Quantification was conducted on a minimum of 50 cells. Data is represented as number of interactions (dots) per cell.

#### Quantification of DNA damage foci

Image quantification was conducted using FIJI v2.0.0 image processing software (Fiji is just ImageJ, available at Fiji.sc, [51]) as previously described [52]. Four to five images were taken of each sample. The DAPI channel was supervised to enable accurate gating of cell nuclei for application to other channels. Size selection (pixel size 2000 to 15,000) and circularity (0.30–1.00) cut-offs were used. Cells on the edge of the image were excluded from the analysis. The number of DNA damage foci per cell nucleus was calculated for approximately 100–200 cells. The information for individual samples was then collated and analysed using the Pandas package in Python 3.5.

### Clinical cohorts

#### Basal-like breast cancer

Samples were stratified into groups as follows: 42 BLBC (negative for ERα, PR, HER2 and positive for CK5/6, CK14 or EGFR), 14 triple-negative non-BLBC (negative for ERα, PR, HER2, CK5/6, CK14 and EGFR) and 26 HER2-Enriched (negative for ERα and PR, positive for HER2). BRCA1-mutation status in this cohort is unknown; however, it is expected to occur in approximately 6.5% of BLBC patients [53]. Samples were obtained under the Garvan Institute ethical approval number HREC 08/145.

#### Kathleen Cuningham Foundation Consortium for research into familial breast cancer (KConfab)

BRCA1-mutant BLBC was sourced from KConfab. A total of 97 BRCA1-mutant BLBC cases were obtained under the Garvan Institute ethical approval number HREC 08/145.

#### Ovarian cancer

A total of 97 HGSOC cases were obtained under the Human Research Ethics Committee of the Sydney South East Area Hospital Service Northern Section (00/115) [54].

## Results

### ID4 interacts with chromatin without regulating transcription

To investigate the molecular function of ID4, we first examined ID4 protein expression and cellular localisation across a panel of breast and ovarian cancer cell lines. Ovarian cancer cell lines were included due to the established molecular similarities between BLBC and HGSOC [5–7]. Furthermore, evidence suggests ID4 may play an important role in the aetiology of both BLBC and HGSOC [11, 55]. As described previously [11], ID4 is predominantly expressed by cell lines of the BLBC subtype (Figure S1A). Variable ID4 protein expression was observed across the ovarian cell lines, and unlike in BLBC, ID4 did not associate specifically with HGSOC, the more aggressive ovarian cancer subtype. Four ID4 expressing models were selected for further analysis: MDA-MB-468 (BLBC), HCC70 (BLBC), HCC1954 (HER2-Enriched) and OVKATE (HGSOC), because these models represent different biological systems with similar levels of ID4 expression.

**Fig. 1:**
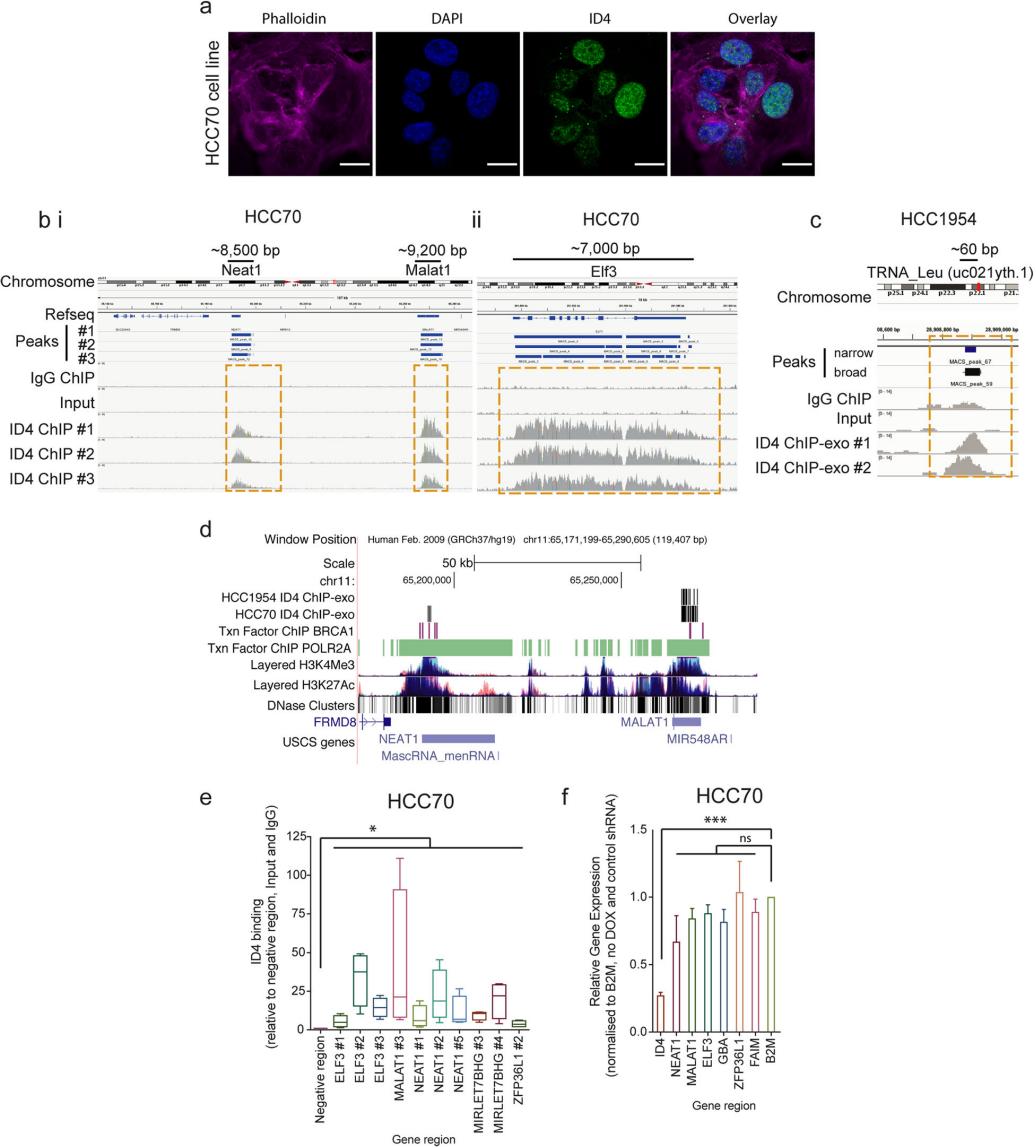
ID4 binds to chromatin in BLBC cell lines and PDX models and does not regulate transcription. **a** Immunofluorescence analysis of ID4 protein expression in the HCC70 BLBC cell line, blue; nuclear marker DAPI, magenta; cytoskeletal marker phalloidin, green; ID4. 20 μm scale. **b** Three technical replicates of ID4 ChIP-seq analysis in HCC70 cell line. IgG ChIP-seq and Input controls are shown for comparison. ID4 binding to (i) the genomic region encoding the long non-coding RNAs NEAT1 and MALAT1 and (ii) the protein coding gene ELF3. **c** ID4 binding to tRNA_Leu (uc021yth.1) in HCC1954 cell line measured by ChIP-exo. Identification of ID4 binding enrichment identified by MACS peak-calling algorithm. Chromosome location, transcription start site (TSS) and Refseq information tracks displayed. Reads have been aligned to the human reference genome Hg19 and peaks called using MACs peak calling algorithm (v2.0.9) [38]. Images contain ChIP-seq coverage data and the peaks called for each ID4 technical replicate and the consensus peaks called for all three ID4 ChIP-seq technical replicate for selected gene regions. ID4 binding is shown in comparison to IgG and Input data for the same region. Data visualised using IGV [56, 57]. Transcription Start Site (TSS) indicated with black arrow. **d** Alignment of ID4 ChIP-exo peaks with DNAse hypersensitivity clusters, Transcription Factor ChIP and histone marks H3K27Ac and H3K4Me3 ChIP-seq at NEAT1 and MALAT1, UCSC Genome Browser. **e** Box and whisker plot of ID4 ChIP-qPCR analysis in HCC70 cells. Multiple primers were designed to tile across the large ID4 binding sites. ID4 binding normalised to input DNA and to a region not bound by ID4 (negative region) and represented as fold-change over IgG control. Ratio paired *t* test, *p < 0.05. Whiskers indicate min to max. *n* = 4. **f** qRT PCR analysis of mRNA transcript expression of ID4-bound genomic regions following depletion of ID4 in HCC70 cells using a lentiviral, doxycycline-inducible short hairpin RNA #2 (SMARTChoice). Data normalised to B2M housekeeping gene and to HCC70 cells treated with vehicle control. One-way ANOVA, multiple comparisons test of control primer B2M with each primer set. ***p < 0.001, ns = non-significant. Error bars represent standard error. *n* = 4

ID4 predominantly localised to chromatin, as evidenced by distinct punctate staining in the nucleus, with a proportion of ID4 staining overlapping with DAPI-low nuclear regions (Fig. 1a), typical of uncondensed euchromatin. Due to this localisation, and a previous report of ID4 associating with enhancer regions in normal mammary epithelial cells [12], chromatin immunoprecipitation and sequencing (ChIP-seq) was used to interrogate whether, and how, ID4 binds chromatin in BLBC. ID4 ChIP-seq was conducted on asynchronous HCC70 cells in three biological replicates and compared with IgG ChIP and an input control. Western blot analysis confirmed successful immunoprecipitation of ID4 (Figure S1B). ChIP-seq data was integrated by removing signal identified in the input and IgG controls and examining the overlap between the three ID4 ChIP-seq replicates. Remarkably, ID4 reproducibly associated with seven sites across the genome (Figure S2A and Table S1). Some of these were focussed, small binding events typical of transcription factor binding. For example, ID4 binding to the transcription start site of *FAIM* and *GBA* (140–230 bp) (Figure S2C) was typical of transcription factor binding peaks. In other locations, ID4 binding was spread over very large regions of DNA, up to 10 kb in length, which is reminiscent of some histone marks, and not transcription factor binding profiles. ID4 bound primarily to gene bodies of the protein-coding genes *ELF3* and *ZFP36L1*, the long non-coding RNAs *NEAT1* and *MALAT1* and the microRNA host-gene *MIRLET7BHG*. The MACS peak-calling algorithm identified multiple peaks per gene for these genes, due to wide spread binding across the region (*ELF3*, *NEAT1*, *MALAT1*, *MIRLET7BHG* and *ZFP36L1*) (Fig. 1b and Figure S2C). This binding is absent from intergenic regions (Fig. 1b).

Due to the large expanses of ID4 binding, ChIP-exonuclease (ChIP-exo) was employed to provide higher resolution mapping of ID4 binding. ChIP followed by exonuclease digestion and DNA sequencing identifies precise transcription factor binding sites with higher resolution than traditional ChIP-seq [38]. ID4 ChIP-exo was conducted in biological duplicate in HCC70 and HCC1954 breast cancer cell lines (Figure S3A and Table S1). Consistent with the initial ChIP-seq experiment, we identified significant enrichment of ID4 binding to the genomic regions encoding NEAT1, MALAT1 and GBA (Figure S3B). Signal was observed at ELF3 and ZFP36L1; however, these regions were not identified through MACS analysis (Figure S3C). Additional ID4 peaks were identified in several transfer RNAs (tRNA) (Fig. 1c) and within the gene bodies of KDM4C and ERRFI1 (Figure S3D). This method was unable to further resolve ID4-chromatin binding information and the data resembled the ChIP-seq data. This suggests that ID4 forms large contiguous and uninterrupted interactions with expanses of chromatin. The ID4-bound chromatin aligned with regions of high transcriptional activity, co-localising with DNaseI hypersensitivity clusters, active histone marks, and BRCA1 and POLR2A transcription factor binding sites (Fig. 1d).

ChIP-qPCR was used to validate ID4 binding in HCC70, HCC1954 and MDA-MB-468 cell lines. Primers to the genes of interest were designed to tile across multiple sites in each gene, with several regions enriched for ID4 binding compared to input and IgG controls (Fig. 1e and Figure S4). ChIP-qPCR validation from independent ChIP experiments validated the findings made from the unbiased ChIP-seq approaches.

To address the specificity of ID4 binding, ChIP-qPCR analysis was conducted on HCC70 cell line modified with the SMARTChoice doxycycline-inducible ID4 short hairpin. Doxycycline treatment resulted in an 87% reduction in ID4 protein expression at 72 h (Figure S5). ID4 knockdown reduced ID4-chromatin binding at a majority of sites (Figure S6b-c), confirming the specific binding of ID4 to these loci.

Although ID proteins are classically thought to be transcriptional regulators, to date, no studies have reported a systematic analysis of ID4 transcriptional targets. Hence, to determine whether ID4 affects transcription in BLBC, we analysed changes in gene expression using RNA-sequencing (RNA-seq) following ID4 depletion using the SMARTChoice model described above. This analysis was also used to identify whether ID4 regulates the expression of genes adjacent to ID4 chromatin interactions. Differential expression analysis revealed limited changes in gene expression following loss of ID4, with only six genes identified as being differentially expressed (Table S2), indicating ID4 does not function as a transcriptional regulator in this model. Loss of ID4 did not affect the expression of the ID4-bound genes identified through ChIP-seq and ChIP-exo analysis, as determined through RNA-seq and qPCR (Fig. 1f). This suggests that ID4 has a transcription-independent role on chromatin.

### ID4 interacts with DNA damage response proteins

**Fig. 2:**
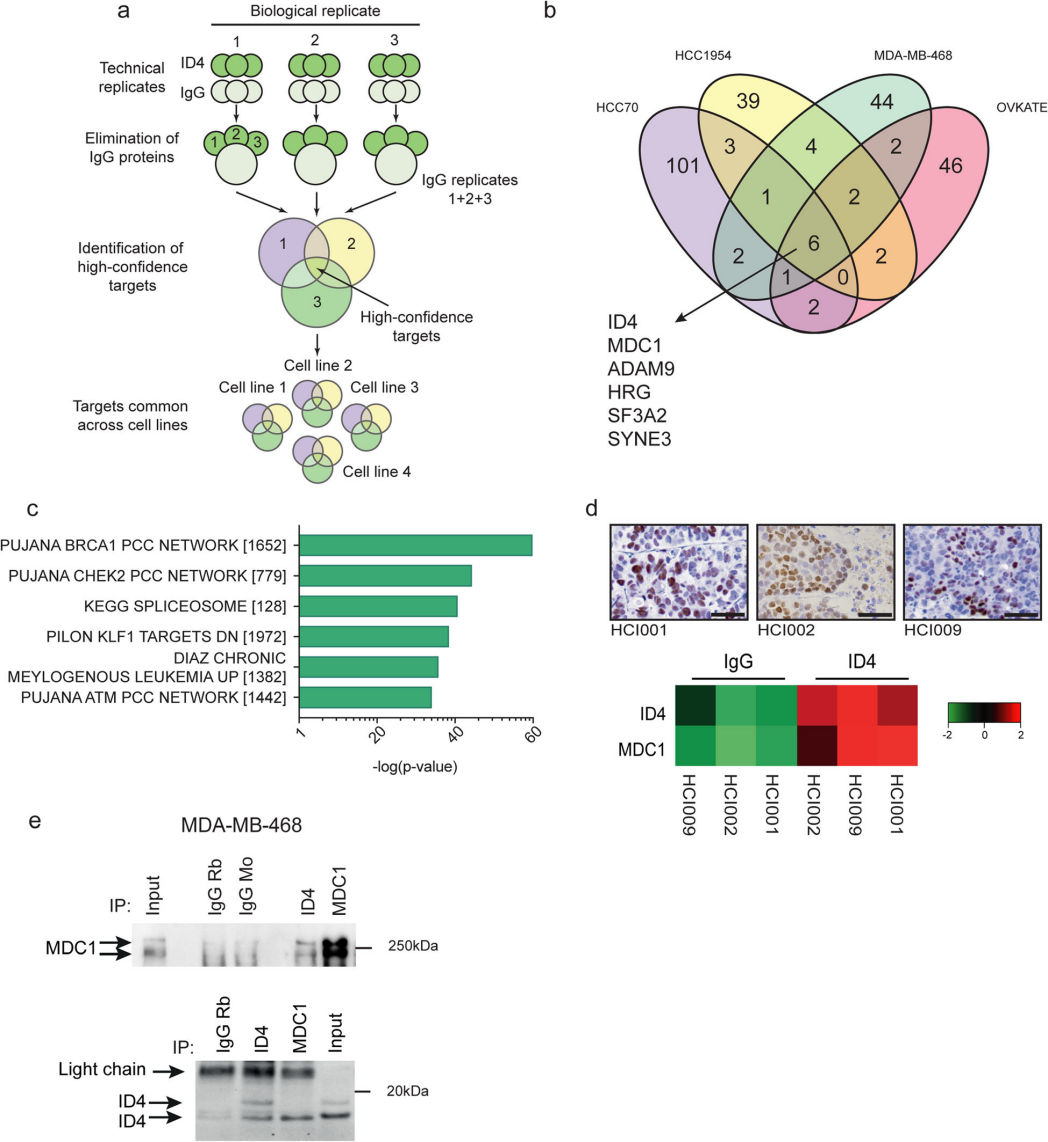
ID4 binds to MDC1 and interacts with the BRCA1 network. **a** Schematic of ID4 and IgG RIME data analysis; ID4 and IgG immunoprecipitations were conducted in technical duplicate or triplicate, and in biological triplicate. All proteins identified in the IgG controls (for each biological replicate) were removed from each of the ID4 IPs as non-specific, background proteins. The remaining proteins were compared across technical replicates to generate a list of medium-confidence proteins that were robustly identified in > 1 ID4 RIME technical replicate. The biological replicates were then compared to generate a list of targets present in > 1 biological replicate in an individual cell line (i.e., > 6 technical replicates conducted over three biological replicates). This resulted in the identification of 22 (HCC70), 21 (HCC1954), 21 (MDA-MB-468) and 30 (OVKATE) proteins. Targets from the different cell lines were compared, identifying a list of ID4 interactors present across multiple cell lines. **b** Venn diagram showing comparison of the high-confidence targets identified in all four cell lines: HCC70, HCC1954, MDA-MB-468 and OVKATE. Common targets across the four cell lines are indicated. Overlap generated using Venny [58]. **c** Top six highest enriched gene sets identified through Gene Set Enrichment Analysis (GSEA) of the ID4 proteome (1106 proteins) identified in (**b**) (all proteins identified in ID4 RIME and not in IgG RIME), compared to C2 (curated gene sets), C4 (computational gene sets), C6 (oncogenic signatures), C7 (immunologic signatures) and H (hallmark gene sets) gene sets. Proteins identified in > 1 technical replicate of ID4 RIME (and not in IgG RIME) were considered in GSEA analysis. **d**, top: Immunohistochemistry analysis of ID4 protein expression across HCI001, HCI002 and HCI009 triple-negative PDX models, 100 μm scale, bottom; SWATH proteomic analysis of ID4 and IgG RIME conducted on HCI001, HCI002, HCI009 PDX models. Heatmap showing quantification of ID4 and MDC1 abundance, both significantly differentially expressed proteins in ID4 RIME compared with control IgG RIME (*p* value < 0.05 and fold change > 2). RIME and SWATH were conducted on the same sample, one biological replicate per tumour. 100 μm scale. **e** Immunoprecipitation was conducted on MDA-MB-468 cells prepared using the RIME protocol. Top: Input control and IgG (mouse and rabbit) compared to IP with anti-ID4 antibodies and MDC1. Western blotting is shown using an independent monoclonal MDC1 antibody. Bottom: Input control and IgG rabbit compared to IP with anti-ID4 antibodies and MDC1. Western blotting is shown using an independent monoclonal ID4 antibody

To understand how ID4 interacts with chromatin in the absence of a known DNA interaction domain, endogenous ID4 was purified from cells using the proteomic technique rapid immunoprecipitation mass spectrometry of endogenous proteins (RIME) [36]. Anti-ID4 and control IgG were used for immunoprecipitations in biological triplicate (each in technical duplicate or triplicate) followed by mass spectrometry in the HCC70, MDA-MB-468, HCC1954 and OVKATE cell lines. Proteins were considered bona fide ID4-binding targets if they appeared in two or more technical and biological replicates and were not present in any IgG controls (Fig. 2a).

RIME analysis identified cell line-unique and common ID4-binding candidates (Fig. 2b and Table S3). In total, 1106 unique proteins were identified across the four cell lines. Gene Set Enrichment Analysis (GSEA) of the putative ID4 binding partners showed enrichment for chromatin (DNA damage and transcription proteins and histones) and RNA-associated gene sets (including RNA splicing and processing factors) (Fig. 2c and Table S3). This compliments the above ChIP-seq data, demonstrating that ID4 interacts with chromatin and nuclear machinery. The BRCA1-PCC network was the most significantly enriched gene set in the ID4 purified proteome (PCC: Pearson correlation coefficient, 101/852 ID4-associated genes were present in the set of 1652 BRCA1-associated genes; *p* = 6.47E−59) (Fig. 2c). This gene set comprises networks controlling breast cancer susceptibility [59]. Other gene sets originating from this previous study [59], including the CHEK2-PCC and ATM-PCC, were also identified as highly enriched, suggesting an association between ID4 and DNA damage repair factors.

Across the entire RIME dataset, only one peptide mapping to a bHLH protein, HEB (also known as TCF12), was identified, in one of nine technical replicates in the OVKATE cell line. We therefore concluded that HLH proteins are not significant ID4 interactors in BLBC and HGSOC.

Six proteins were commonly identified in all four cell lines: ID4, mediator of DNA damage checkpoint 1 (MDC1), disintegrin and metalloprotease domain 9 protein (ADAM9), Histidine Rich Glycoprotein (HRG), splicing factor 3a subunit 2 (SF3A2) and spectrin repeat containing nuclear envelope family member 3 (SYNE3). The most highly enriched protein, MDC1, was the only protein to be identified in all RIME technical and biological replicates in all four cell lines. MDC1 has many cellular functions, including cell cycle control and transcription [60, 61], yet is best characterised for its role in the DNA damage response, where it associates with DNA [62]. Of the other proteins identified, ADAM9 and HRG affect cell motility, SYNE3 is important in cytoskeletal and nuclear organisation [63] and SF3A2 is required for pre-mRNA splicing and the generation of alternative transcripts [64]. ID4 was previously suggested to bind to mutant p53 and SRSF1 [65, 66], yet no evidence was found of these associations in our analysis.

To validate these findings in a more clinically relevant setting, we conducted ID4 RIME in patient-derived xenograft (PDX) models of triple-negative breast cancer [49]. Three ID4-expressing models (HCI001, HCI002, HCI009) were selected for RIME analysis based on detectable expression of ID4 (Fig. 2d). ID4 and MDC1 were the only targets identified in ID4 RIME in all three xenograft models and absent in the IgG RIME (see Table S3 for full list of proteins identified in PDX models). This discovery proteomics was supported by quantitative mass spectrometry analysis of the models using the sensitive label-free quantitative proteomic method termed SWATH (Sequential Window Acquisition of all THeoretical mass spectra) [67]. This analysis showed a high abundance of both ID4 and MDC1 in all three PDX models (Fig. 2d). Due to the robust identification of MDC1 in the four cell lines and the subsequent validation of this interaction in three PDX models, we focused further studies on the functional importance of the ID4-MDC1 relationship.

ID4-MDC1 binding was validated using independent methods including immunoprecipitation with an anti-ID4 antibody and anti-MDC1 antibody, followed by western blotting with an independent monoclonal ID4 or a MDC1 antibody (Fig. 2e). This co-immunoprecipitation experiment confirmed the ID4-MDC1 interaction in MDA-MB-468 cells.

### ID4 interacts with DNA repair proteins at fragile chromatin sites in a DNA damage-dependent manner and regulates DNA damage signalling

**Fig. 3:**
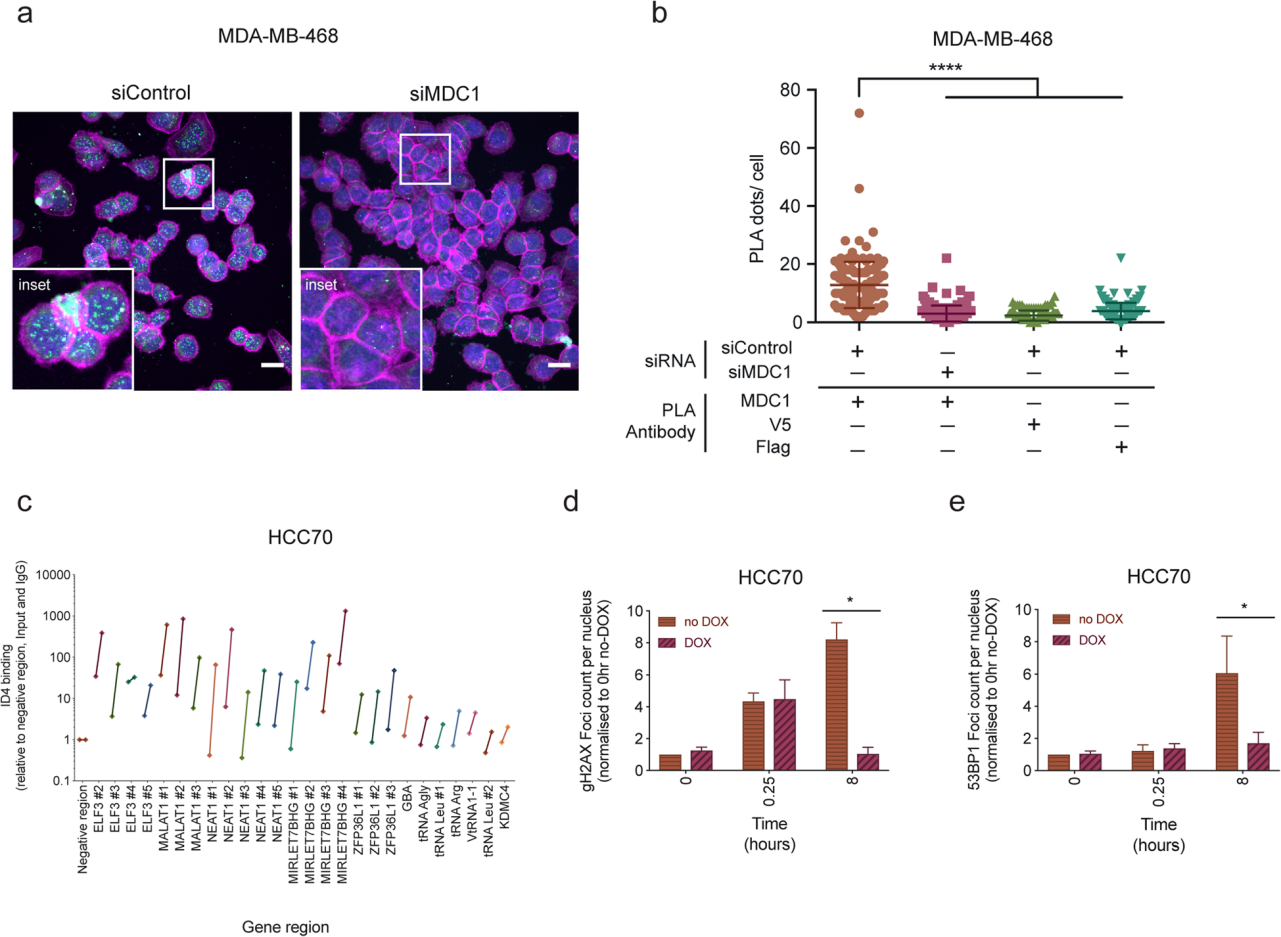
ID4 interacts with DNA damage proteins at fragile chromatin sites in a DNA damage-dependent manner. **a** Representative proximity ligation assay (PLA) confocal images of ID4 and MDC1 interactions in MDA-MB-468 cells. siRNAs targeting MDC1 (siMDC1) and a scrambled control (siControl) used to show specificity of the assay. PLA foci (green), DAPI (blue) and phalloidin (magenta). 20 μm scale. **b** Graph showing quantification of PLA interactions between ID4 and MDC1 in cells treated with siControl and siMDC1. Data shown in comparison to the interaction between ID4:V5 and ID4:FLAG treated with siControl. 50–100 cells captured per condition. Quantification of interactions (number of dots/ cell). One-way ANOVA, multiple comparisons test. ****p < 0.0001. Error bars represent standard deviation. **c** ChIP-qPCR analysis of ID4 binding in untreated HCC70 cells compared to cells treated with ionising radiation (5 Gy with 5 h recovery time prior to fixation). ID4 binding normalised to negative control region, input DNA and IgG control. Data for each gene region is shown for both untreated (left) and ionising radiation-treated (right) cells, connected by a line. Representative of two independent experiments. Quantification of a time course of **d** γH2AX and **e** 53BP1 DNA damage foci formation following ionising radiation. ID4 was depleted from cells using the SMARTChoice inducible shRNA system following treatment with doxycycline for 72 h prior to treatment with 5 Gy ionising radiation at time 0 and allowed to recover for 0.25 and 8 h prior to analysis. Four to five images were taken for each condition; 100–200 cells in total. Number of foci per cell nucleus was calculated using FIJI by ImageJ (Schindelin et al., 2012), and samples were then collated and analysed using the Pandas package in Python 3.5. Data is normalised to 0 h, no DOX, no IR time point. *n* = 3–5, Student’s *t* test, *p < 0.05. Error bars represent standard error

To further explore the interaction between ID4 and MDC1, we used proximity ligation assays (PLA), a quantitative measure of protein interaction in situ, and confirmed binding of ID4 with MDC1 in MDA-MB-468 cell line. This interaction occurred predominantly in the cell nucleus (Fig. 3a). Depletion of MDC1 using siRNA resulted in reduction of the PLA signal to control levels (Fig. 3b). PLA analysis was also used to test the association of ID4 with γH2AX, as it is required for signalling double-stranded DNA breaks and with BRCA1, as it is a critical mediator of the homologous recombination (HR) repair pathway. PLA analysis identified enrichment of ID4 binding with MDC1, γH2AX and BRCA1 above control levels in HCC70 and with MDC1 in OVKATE cell lines (Figure S7).

To more closely examine the correlation between DNA damage and ID4 complex association with chromatin, we used ionising radiation (IR) to induce DNA damage in cells. Irradiation markedly increased ID4 chromatin binding, measured by ChIP-qPCR; up to 160-fold compared to undamaged controls at the NEAT1 gene, and 5–70-fold at ELF3, MALAT1, MIRLET7BHG, ZFP36L1, GBA and various tRNAs (Fig. 3c and Figure S8). This indicates that ID4 binding is specific and induced by DNA damage. ChIP-qPCR for MDC1 similarly showed induced binding to a majority of these sites in damaged cells (Figure S8). Taken together, these results suggest a positive correlation between DNA damage and ID4-chromatin binding.

To investigate the functional role for ID4 in the DDR, DNA damage was induced using IR and damage sensing was monitored over time. Cells were treated with doxycycline to deplete ID4 for 72 h (point of maximal ID4 knockdown) prior to treatment with ionising radiation. Cells were fixed at 0, 15 min and 8 h following ionising radiation, to examine the DNA damage response over time. As previously reported [68], the number of γH2AX foci in control cultures increased at 15 min post-IR and remained high at 8 h (Fig. 3d), while the number of 53BP1 foci increased at 8 h following ionising radiation (Fig. 3e). Upon ID4 depletion, formation of γH2AX and 53BP1 foci was significantly reduced at 8 h post-IR. This suggests that in the absence of ID4, the DNA was either repaired more efficiently, or alternatively that the DNA damage response was not triggered properly.

### ID4 genetically interacts with *BRCA1* mutation

The data presented earlier suggests an interaction between ID4 function and DNA damage pathways. Patients with germline *BRCA1* mutations are at elevated risk of BLBC and HGSOC [3]. While BRCA1-associated cancers often undergo *BRCA1* loss of heterozygosity (LOH), recent evidence suggests that mutation and copy number change of additional genes, such as *TP53*, often occur prior to, and may be required for, loss of the wildtype *BRCA1* allele [69]. We therefore assessed whether there is evidence for copy number change of *ID4* in (i) sporadic disease or (ii) in the context of familial breast cancer driven by germline *BRCA1* mutation.

**Fig. 4:**
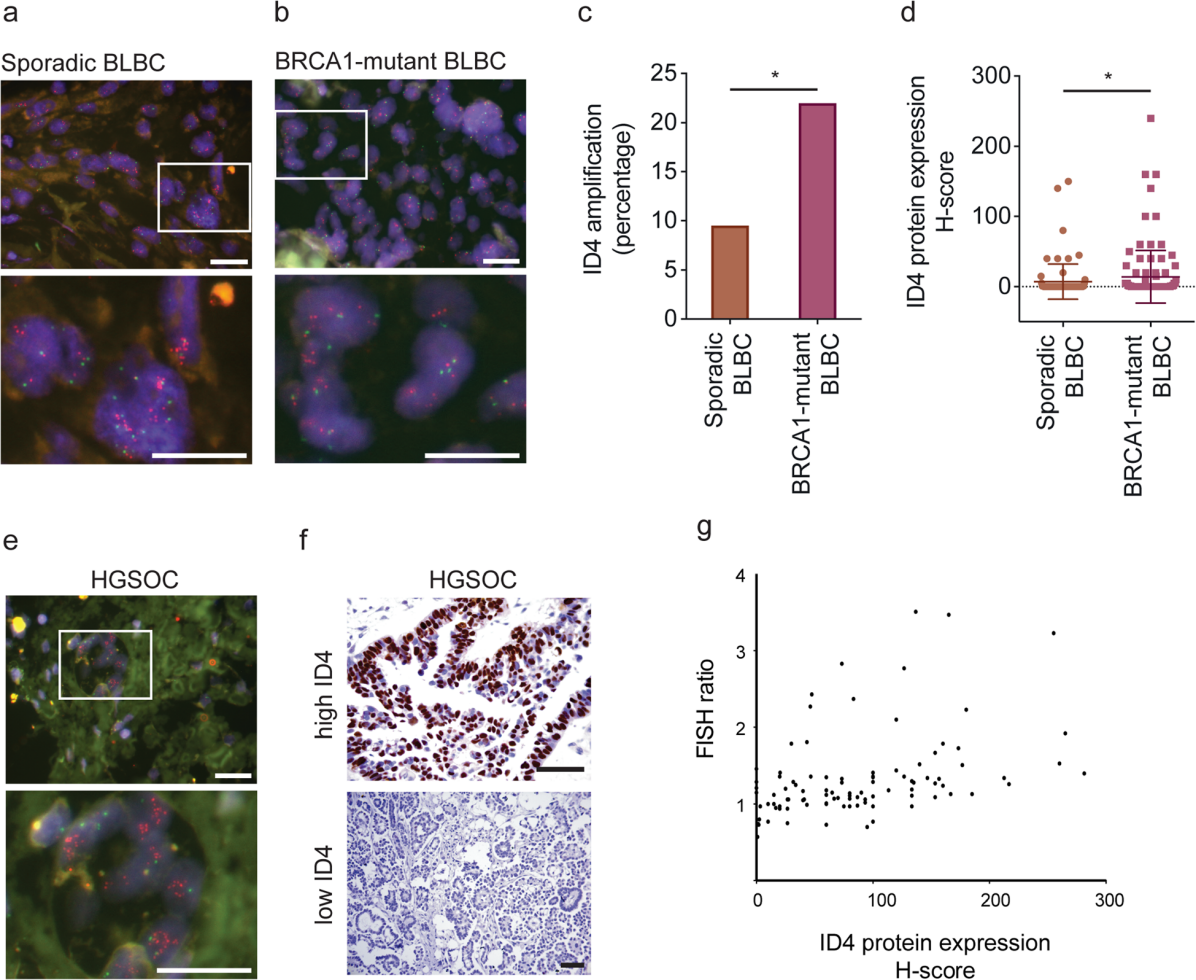
ID4 is selectively amplified in cancers arising in germline BRCA1 mutation carriers. **a** ID4 amplification in sporadic and familial BLBC. Example images of fluorescent in situ hybridisation (FISH) analysis of ID4 (red) and CEP6 centromeric control (green) copy number aberrations in a cohort of 42 sporadic BLBCs. 10 μm scale. **b** Example images of FISH analysis of ID4 (red) and CEP6 centromeric control (green) copy number aberrations in a cohort of 97 BRCA1-mutant BLBCs. 10 μm scale. **c** Percentage of ID4 amplified cases in sporadic and BRCA1-mutant BLBCs presented in **a** and **b**. *p < 0.05 (two-sample *t* test). **d** Immunohistochemical analysis of ID4 protein expression in the sporadic and BRCA1-mutant BLBCs presented in **a** and **b**. ID4 protein quantified using the *H*-score method. *H-*score = intensity of staining (on a scale from 0 to 3) × percentage of cells positive. *p* = 0.007 (Mann-Whitney two-tailed test). **e** Example images of FISH analysis of ID4 (red) and CEP6 centromeric control (green) copy number aberrations in a cohort of 97 high-grade serous ovarian cancers. 10 μm scale. **f** Immunohistochemistry staining for ID4 in HGSOC. Example images of ID4 high expressing (top) and ID4 low expressing (bottom) HGSOC. **g** ID4 H-score compared with corresponding *ID4*:*CEP6* FISH ratio. Assuming non-Gaussian distribution, *H*-score and FISH correlated with a value of *r* = 0.457 and Spearman correlation *p* ≤ 0.0001

ID4 is reportedly amplified in sporadic BLBC [5, 6, 55]; however, these results are based on small cohorts and imprecise array Comparative Genomic Hybridisation (aCGH) techniques, both of which affect accurate determination of amplification frequency. To definitively quantify *ID4* amplification frequencies, we used fluorescent in situ hybridisation (FISH) in a clinical diagnostic laboratory, a highly specific approach for analysing copy number. FISH was applied to a discovery cohort of 82 sporadic oestrogen receptor-negative invasive ductal carcinomas: composed of 42 BLBC (negative for ERα, PR, HER2 and positive for CK5/6, CK14 or EGFR), 14 triple-negative non-BLBC (negative for Erα, PR, HER2, CK5/6, CK14 and EGFR) and 26 HER2-Enriched (negative for Erα and PR, positive for HER2) samples [11]. *ID4* amplification was exclusive to the BLBC subtype with an amplification frequency of ~ 10% (4/42 cases), and amplification correlated with ID4 protein expression (*r* = 0.265, *p* = 0.0088) (Fig. 4a and Figure S9).

We also examined whether ID4 is amplified in sporadic HGSOC. Analysis of 97 sporadic HGSOC cases [54] showed a comparable rate of *ID4* amplification relative to sporadic BLBC of ~ 10% (10/97 in cases of HGSOC) (Fig. 4e, g). However, these frequencies are lower than that published using Array CGH [5, 6], potentially suggesting that aCGH methods overestimate amplification frequencies or that these are distinct clinical cohorts. ID4 protein expression in HGSOC correlated with amplification (*r* = 0.457 and *p* < 0.0001) (Fig. 4f, g).

To determine potential genetic interactions between *ID4* and *BRCA1*, *ID4* copy number and protein expression was evaluated in 97 familial BLBCs from patients with known germline *BRCA1* mutations and compared to the 42 unselected BLBC (which are not expected to frequently carry *BRCA1* germline mutations) described above. *ID4* was amplified at twice the frequency in *BRCA1*-mutant BLBC (~ 22%, 21/97 cases) compared to sporadic BLBC (~ 10%, 4/42 cases), indicating a selection for *ID4* amplification in cancers arising in patients carrying a mutant *BRCA1* allele (Fig. 4b, c). ID4 protein expression was also significantly higher in the *BRCA1*-mutant BLBC compared to the sporadic BLBC cohort (Fig. 4d). These data provide further evidence for an important role for ID4 in BLBC and HGSOC aetiology.

## Discussion

By elucidating the drivers and dependencies of BLBC, we aim to improve our understanding of this complex, heterogeneous disease, potentially leading to the identification of novel targets and therapeutics. Previous work from our group and others has shown that ID4 acts as a proto-oncogene in BLBC [11, 14–21]. ID4-positive BLBC have a very poor prognosis, and depletion of ID4 reduced BLBC cell line growth in vitro and in vivo [11], suggesting that ID4 controls essential, yet unknown intrinsic pathways in BLBC.

We have conducted the first systematic mapping of the chromatin interactome of ID4 in mammalian cells. Using ChIP-seq, we have identified novel ID4 binding sites within the BLBC genome. ID4 bound to large regions of chromatin, up to 10 kb in length, at a very small number of loci, suggesting that ID4 is not binding as part of a conventional transcriptional regulatory complex. This conclusion is supported by the observation that ID4 knockdown did not affect gene expression at these loci. The regions identified through ChIP-seq were typically the bodies of genes that are highly transcribed and mutated in cancer [70, 71], characteristics of fragile, DNA damage-prone sites. Interestingly, these sites primarily encode non-coding RNA, including lncRNA, microRNA precursors and tRNA. The lncRNA NEAT1 and MALAT1 are some of the most abundant cellular RNAs and the genes encoding them undergo recurrent mutation in breast cancer [72]. Furthermore, they are upregulated in ovarian tumour cells and are associated with higher tumour grade and stage and in metastases [73]. The genomic loci encoding tRNAs are also highly transcriptionally active, co-localising with DNaseI hypersensitivity clusters and transcription factor binding sites, including BRCA1 and POLR2A [74]. Transcriptional activity is highly stressful and associated with genomic stress and DNA damage [70]. Binding of ID4 to these sites was increased upon DNA damage. Together, these data suggest that ID4 binds preferentially to sites of active transcription and DNA damage, consistent with its interaction with MDC1.

We have conducted the most systematic and unbiased proteomic analysis of binding partners for any ID family member. RIME revealed hundreds of ID4 interacting proteins, which were highly enriched for BRCA1-associated proteins. Five novel proteins were found to interact with ID4 with high confidence in all 4 models examined, namely MDC1, ADAM9, HRG, SF3A2 and SYNE3. These warrant further investigation to examine the role they may have in the mechanism of action of ID4. Interestingly, no bHLH proteins were reproducibly found in complex with ID4, although they are the canonical ID binding partners in certain non-transformed cells [22–26]. This is unlikely to be a technical artefact, as ID4-bHLH interactions are readily detected in non-transformed mammary epithelial cells using the same method (H. Holliday et al; Unpublished data). Rather, ID4 may have alternate binding partners as a consequence of downregulation of bHLH proteins in BLBC (H. Holliday et al; Unpublished data).

ID4 has been demonstrated to be a member of a ribonucleoprotein complex along with mutant p53, SRSF1 and lncRNA MALAT1 [66]. This complex promotes splicing of VEGFA pre-mRNA, which signals in a paracrine manner to macrophages and ultimately results in tumour angiogenesis [65, 66, 75]. p53 and SRSF1 were not identified in our RIME analysis. This disparity may be the consequence of methodological differences such as fixation, cell lysis conditions and detection techniques. It is possible that ID4 is a member of a large complex encompassing several splicing factors (including SF3A1 and SRSF1). The observations that ID4 interacts with both the MALAT1 gene, as well as MALAT1 lncRNA itself [66], suggests that ID4 and MALAT1 function are intimately linked and warrants further investigation.

MDC1, the most reproducible binding partner of ID4 identified through RIME analysis, is recruited to sites of DNA damage to amplify the phosphorylation of H2AX (to form γH2AX) and recruit downstream signalling proteins [62]. Deficiency in MDC1, much like BRCA1, results in hypersensitivity to double-stranded DNA breaks [76]. MDC1 interacted with many of the sites of chromatin interaction by ID4 in a DNA damage-dependent manner. We also find ID4 in close proximity of known MDC1 interactors, including BRCA1 and γH2AX. These data suggest a model in which ID4 associates with the DNA damage repair apparatus at sites of genome instability or damage via its interaction with MDC1. MDC1 was also recently found to associate with ID3, suggesting that this is a conserved feature of ID proteins [77]. How MDC1 binds ID4 is unknown; however, a quasi HLH domain [78] structure within MDC1 may enable interaction with the HLH domain of ID4. As complex feedback mechanisms govern the DNA damage response, further investigation is required to determine whether ID4 association with MDC1 ultimately promotes or impedes DNA repair.

BRCA1 has proposed functions as a transcription factor controlling differentiation in non-transformed cells [74, 79], but a primary role in DNA repair in cancer [9]. Similarly, ID4 is an important regulator of stemness in the developing mammary gland, acting to inhibit differentiation [11, 12]. However, the results presented here have uncovered an unexpected role for ID4 in the DNA damage response in BLBC, suggesting a similar dichotomy of function to BRCA1, that is, primarily regulating transcription during development whilst predominantly regulating the DNA damage response in cancer. Transcription is a stressful cellular process causing significant DNA damage and repair [70]. Thus, a role for transcription factors in localising DNA damage machinery to chromatin may be an important cellular capability.

Mutations in BRCA1 predispose carriers predominantly to cancers of the breast and ovaries (mostly BLBC and HGSOC), though the mechanism driving tumorigenesis in these patients is still unclear. While many BRCA1-associated cancers undergo LOH for the wildtype BRCA1 allele, many acquire other genomic ‘hits’ prior to this event, which may be required for subsequent LOH [80]. In addition to reporting a biochemical interaction between ID4 and MDC1, we also show a novel genetic interaction with BRCA1, in that *ID4* is amplified at twice the frequency in *BRCA1*-mutant BLBC compared to sporadic BLBC, making it one of the most frequently amplified genes in that disease. A caveat of this finding is that other cancer-associated genes, such as *E2F3*, are located adjacent to *ID4* at Chr6q22 and so may also be a target of the amplification event.

Further work is required to understand the drivers of ID4 amplification and its contribution to DNA repair; however, at least 2 scenarios are possible. In the first, ID4 acts to suppress DNA repair proficiency and so cooperates with *BRCA1* haploinsufficiency to promote genomic instability and tumourigenesis. This is consistent with the positive correlation between ID4 expression and ‘BRCAness’ [15], a defect in BRCA1 function in the absence of germline *BRCA1* mutations [81]. However, perhaps more likely is the opposing scenario, that *ID4* amplification and overexpression promote DNA damage repair, consistent with our observations of ID4 association with MDC1 at fragile sites and the ongoing requirement for ID4 in BLBC proliferation that we previously reported [11]. In the case of BRCA1-associated breast cancer, *ID4* amplification, like *TP53* mutation, may be permissive for subsequent *BRCA1* LOH which is otherwise lethal [69], explaining the high frequency of *ID4* amplification in familial cancers. Further resolving the function of ID4 in BLBC will require detailed biochemical analysis of DNA repair functional assays, genetic studies with ID4 knockout cells or animals and access to a large cohort of familial breast cancers with detailed gene copy number, BRCA1 mutation and methylation data.

## Conclusions

In summary, in this integrated analysis of ID4 function in BLBC and HGSOC, we show that ID4 localises to DNA at sites of active transcription and DNA damage, bridged through its biochemical interaction with the DNA damage response machinery, namely MDC1. Rather than regulating transcription at these sites, our data points to a role of ID4 in the DNA damage response. Finally, we establish a genetic interaction between *BRCA1* and *ID4*, where *ID4* is amplified at twice the frequency in *BRCA1-*mutant BLBC compared to sporadic disease. Though further studies our required to dissect the precise involvement of ID4 in the DNA damage response, these novel insights expand upon our current understanding of the mechanism by which ID4 drives malignancy in breast and ovarian cancer.

## Supplementary information

**Additional file 1: Figure S1.** Analysis of ID4 protein expression across a panel of breast and ovarian cell lines. (A) Western blotting analysis of ID4 protein expression across a panel of breast (top) and ovarian (bottom) normal and cancer cell lines. A rabbit monoclonal antibody specific to ID4 (Biocheck, BCH-9/82–12) was used for detection [11]. Two isoforms of ID4 are detectable across the panel. β-Actin is shown as a loading control. Modifications to images indicated with vertical black line. Cell lines selected for further analysis are indicated with a black square. Breast cancer subtypes and ovarian cancer histotypes indicated [82, 83]. (B) Western blot validation of RIME protocol. MDA-MB-468 cells were processed using the RIME protocol. Antibodies targeting ID4 (pooled polyclonal antibodies) and IgG (rabbit species matched control) control were used for immunoprecipitation. Western blot analysis for ID4 protein expression, using an independent ID4 monoclonal antibody, following immunoprecipitation. **Figure S2.** ID4 ChIP-sequencing analysis of HCC70 cell line identifies reproducible ID4-chromatin binding sites. (A) Table summarising three biological replicates of ID4 ChIP-seq analysis in HCC70 cell line. (B) 10,000 bp resolution image of ID4 ChIP-seq technical replicate #1 binding to MALAT1 gene. Red sequencing reads are aligned to the positive strand (5′ - 3′), and blue to the negative strand of DNA (3′ - 5′). ID4 binding to (C) left to right: GBA, FAIM, MIRLET7BHG, NEAT1 and ZFP36L1. The chromosomal location, size of the gene and Refseq, human reference genome, are displayed at the top of the image. Reads have been aligned to the human reference genome Hg19 and peaks called using MACs peak calling algorithm (v2.0.9) [38]. Images contain ChIP-seq coverage data and the peaks called for each ID4 technical replicate and the consensus peaks called for all three ID4 ChIP-seq biological replicate for selected gene regions. ID4 binding is shown in comparison to IgG and Input data for the same region. Data visualised using IGV [56, 57]. Transcription Start Site (TSS) indicated with black arrow. **Figure S3.** ID4 ChIP-exonuclease sequencing analysis of HCC70 and HCC1954 cell lines reproduces ChIP-seq analysis. (A) Table summarising ChIP-exonuclease sequencing analysis of ID4 and IgG binding events in HCC70 and HCC1954 breast cancer cell lines. ID4 binding normalised as for ChIP-seq analysis. ChIP-exo analysis of the HCC70 and HCC1954 breast cancer cell lines showing ID4 binding to NEAT1, MALAT1 and GBA (B), ZFP36L1 and ELF3 (C), and KDM4C and ERRFI1 (D). Reads have been aligned to the human reference genome Hg19 and peaks called using MACs peak calling algorithm (v2.0.9) [38]. Images contain ChIP-exo coverage data and the peaks called for each ID4 technical replicate and the consensus peaks called for both ID4 ChIP-exo technical replicate for selected gene regions. ID4 binding is shown in comparison to IgG and input data for the same region. Data visualised using IGV [56, 57]. The chromosomal location and size of the gene are displayed at the top of the image. Below this, Refseq, human reference genome, displays the gene corresponding to particular genomic loci. Transcription Start Site (TSS) indicated with black arrow. **Figure S4.** Validation of ID4 binding to specific loci in HCC70, MDA-MB-468 and HCC1954 cell lines. (A) Schematic of primer binding across ELF3 gene region. Primers 1–5 are scattered along the length of the ELF3 gene ID4. ChIP-qPCR analysis in (B) HCC70 (*n* = 2–8), (C) MDA-MB-468 (n = 2) and (D) HCC1954 (n = 2) cells. Multiple primers were designed to tile across the large ID4 binding sites. ID4 binding normalised to input DNA and to a region not bound by ID4 (negative region) and represented as fold-change over IgG control. 1-Way-ANOVA of ID4 binding across all primers compared to negative region: HCC70 *p* = 0.0001, MDA-MB-468 *p* = 0.549, HCC1954 *p* = 0.519. **Figure S5.** ID4 knockdown using SMARTChoice inducible lentiviral knockdown system. Representative western blot showing ID4 and β-Actin protein expression in wild-type HCC70 cell line and HCC70 cells stably transfected with SMARTChoice vectors containing doxycycline-inducible shRNAs targeting ID4 (shID4 #1, #2 and #3) or non-targeting control region (shNTC). Cells treated with and without DOX for 72 h. (B) Western blotting densitometry quantification of ID4 protein expression across six biological replicates of ID4 knockdown following treatment with and without DOX for 72 h. ID4 expression normalised to β-ACTIN and no DOX control. Error bars measure standard error, *n* = 4–6. (C) qRT-PCR analysis of ID4 RNA expression matched to cells in (B). Data normalised to B2M housekeeping gene and no DOX control. Error bars measure standard error, n = 4–6. Student’s t-test compares shNTC with the other modified cell lines. ** *p* < 0.01, **** *p* < 0.001. **Figure S6.** Validation of specificity of ID4-chromatin binding using the SMARTChoice ID4 knockdown model. (A) Western blot showing ID4 and β-Actin protein expression in HCC70 cells stably transfected with shID4 #2 SMARTChoice vector treated with and without doxycycline for 72 h. (B) ID4 ChIP-qPCR analysis of cells from (A). ID4 binding normalised to input DNA, to a region not bound by ID4 (negative region) and to the IgG control. Data represented as fold-change over untreated cells. (C) replicate of ID4 ChIP-qPCR analysis of HCC70 cells stably transfected with shID4 #2 SMARTChoice vector treated with and without doxycycline for 72 h. **Figure S7.** Proximity ligation assay analysis identifies constitutive and DNA damage-inducible binding of ID4 to DNA damage proteins. PLA analysis of ID4 association with FLAG, MDC1, BRCA1 and γH2AX in (A) HCC70 and (B) OVKATE cell line. Analysis is representative of approximately 150–200 cells of pooled from two independent experiments. 1-way Anova *p* = 0.098. B, right; example image of ID4 and MDC1 PLA. Quantification of interactions presented on right (number of dots/ cell). 10 μm scale. **Figure S8.** Ionising radiation increases DNA damage foci formation and localisation of MDC1 to chromatin. Induction of DNA damage using ionising radiation measured using immunofluorescence analysis in MDA-MB-468 cells for γH2AX (top) and MDC1 (bottom) foci formation (5Gy with 5 h recovery time prior to fixation). Example images on left, quantification of foci on right. Four to five images were taken for each condition; 50–100 cells. 20 μm scale (B) Second replicate of HCC70 ID4 ChIP-qPCR. (C) ChIP-qPCR analysis of MDC1 positive control binding following ionising radiation DNA damage induction. Data normalised as for Fig. 3c, with data shown as fold-change compared to no-IR, *n* = 1. **Figure S9.** ID4 is overexpressed and amplified at a higher frequency in BRCA1-mutant BLBC. (A) Graph of ID4 protein expression measured by H-score. Contains 97 BRCA1-mutant BLBC patients. (B) ID4 protein expression measured by immunohistochemistry H-score compared with corresponding *ID4:CEP6* FISH ratio. Assuming non-Gaussian distribution, H-score and FISH correlated with a value of r = 0.265 and Spearman correlation *p* value of < 0.00881.

**Additional file 2: Table S1.** ChIP-seq and ChIP-exo MACS peaks.

**Additional file 3: Table S2.** ID4 RNA-seq differentially expressed genes.

**Additional file 4: Table S3.** Putative ID4 interaction proteins identified by RIME.

**Additional file 5: Table S4.** ChIP-qPCR primers.

## Data Availability

The datasets used and/or analysed during the current study are included as supplementary information files.

## Abbreviations

BLBC: Basal-like breast cancer
BRCA1: Breast and ovarian cancer susceptibility gene
ChIP-seq: Chromatin immunoprecipitation followed by DNA sequencing
FISH: Fluorescent in situ hybridisation
GSEA: Gene Set Enrichment Analysis
HGSOC: High-grade serous ovarian cancer
HLH: Helix-loop-helix
HR: Homologous recombination
ID4: Inhibitor of differentiation 4
IR: Ionising radiation
LOH: Loss of heterozygosity
MDC1: Mediator of DNA damage checkpoint 1
PDX: Patient-derived xenograft
PLA: Proximity ligation assay
RIME: Rapid immunoprecipitation and mass spectrometry of endogenous proteins
SWATH: Sequential Window Acquisition of all THeoretical mass spectra
