# Supplementary material for: Proteogenomic analysis of Inhibitor of Differentiation 4 (ID4) in basal-like breast cancer
Source: Breast Cancer Res. 2020 Jun 11;22:63. doi: 10.1186/s13058-020-01306-6 (PMC7291584; doi:10.1186/s13058-020-01306-6)

Supplementary Figure 1

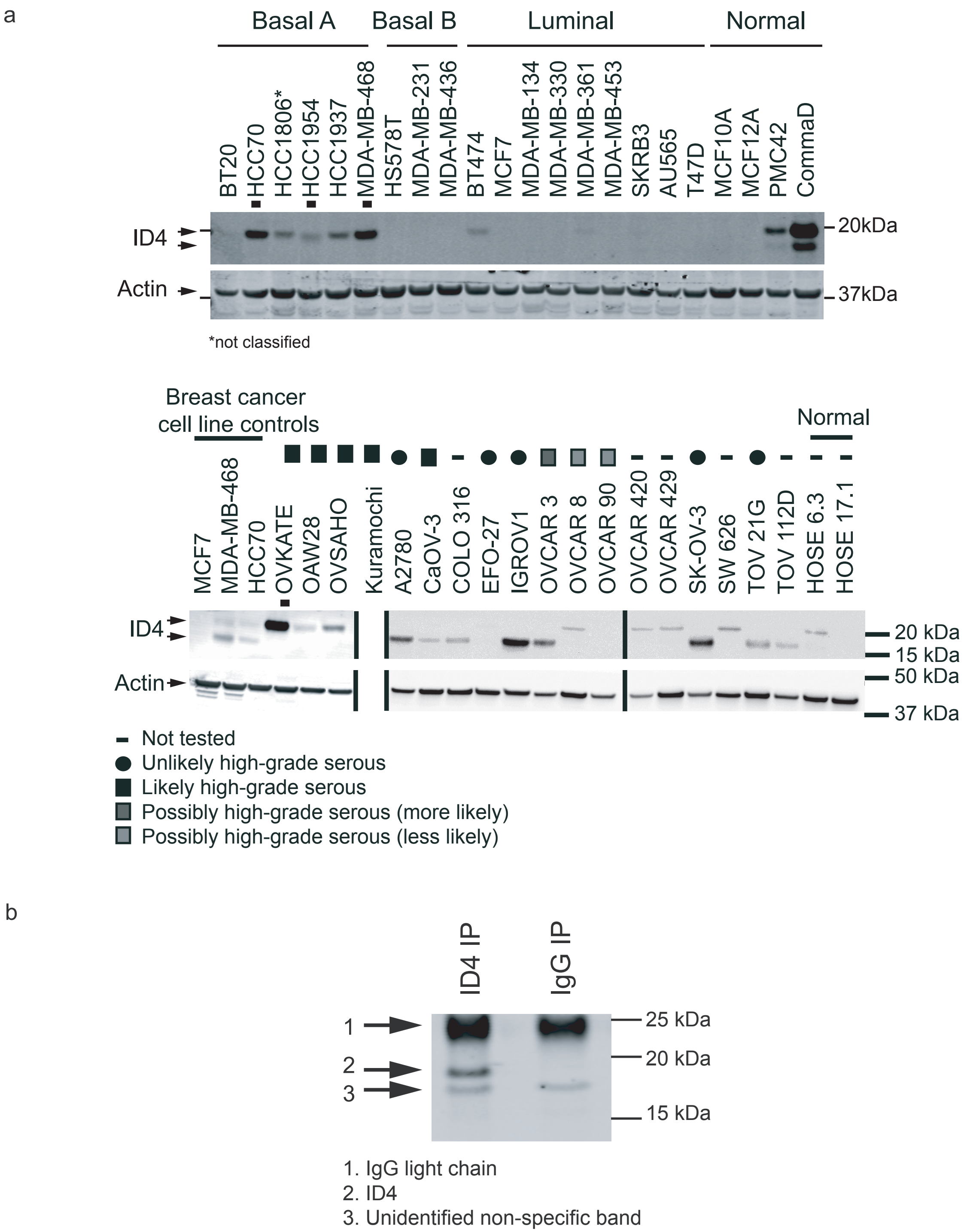

Supplementary Figure 2

a

Cell line	ID4 ChIP replicate	Peaks (after IgG removal)	Consensus peaks	Peaks present in unique genes
HCC70	1	81	46	7
	2	63		
	3	73		

b

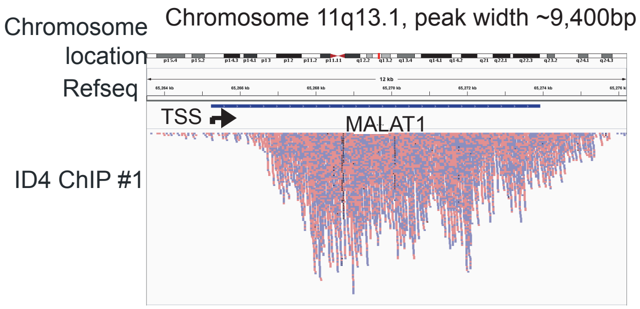

c

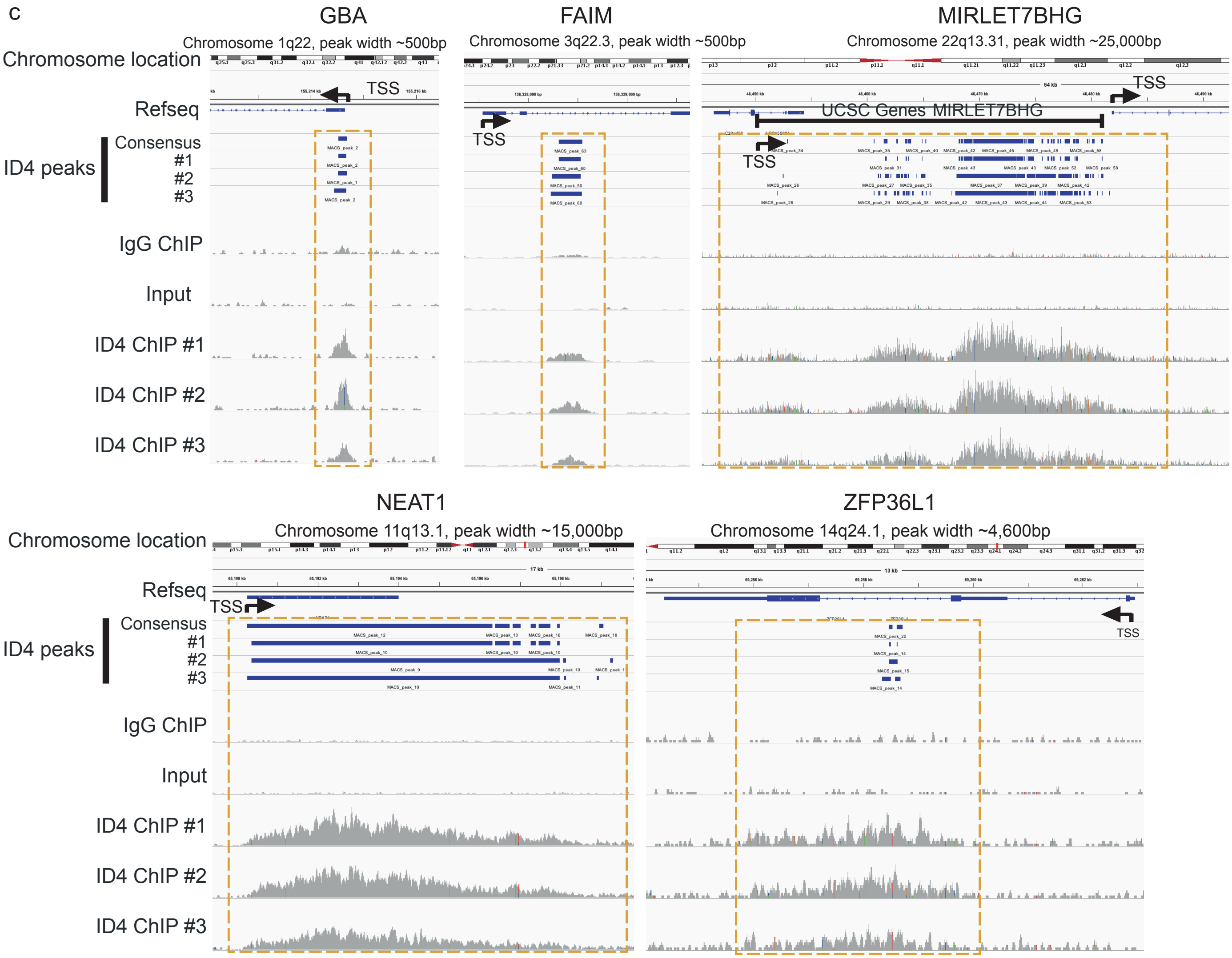

Supplementary Figure 3

a

Cell line	ID4 ChIP replicate	Peaks (after IgG removal)	Peaks (Replicate consensus)	Peaks (after IgG and ENCODE removal)	Peaks (present in unique genes)
HCC70	ID4 replicate 1	154	76	35	16
	ID4 replicate 2	169			
HCC1954	ID4 replicate 1	76	40	37	20
	ID4 replicate 2	64			

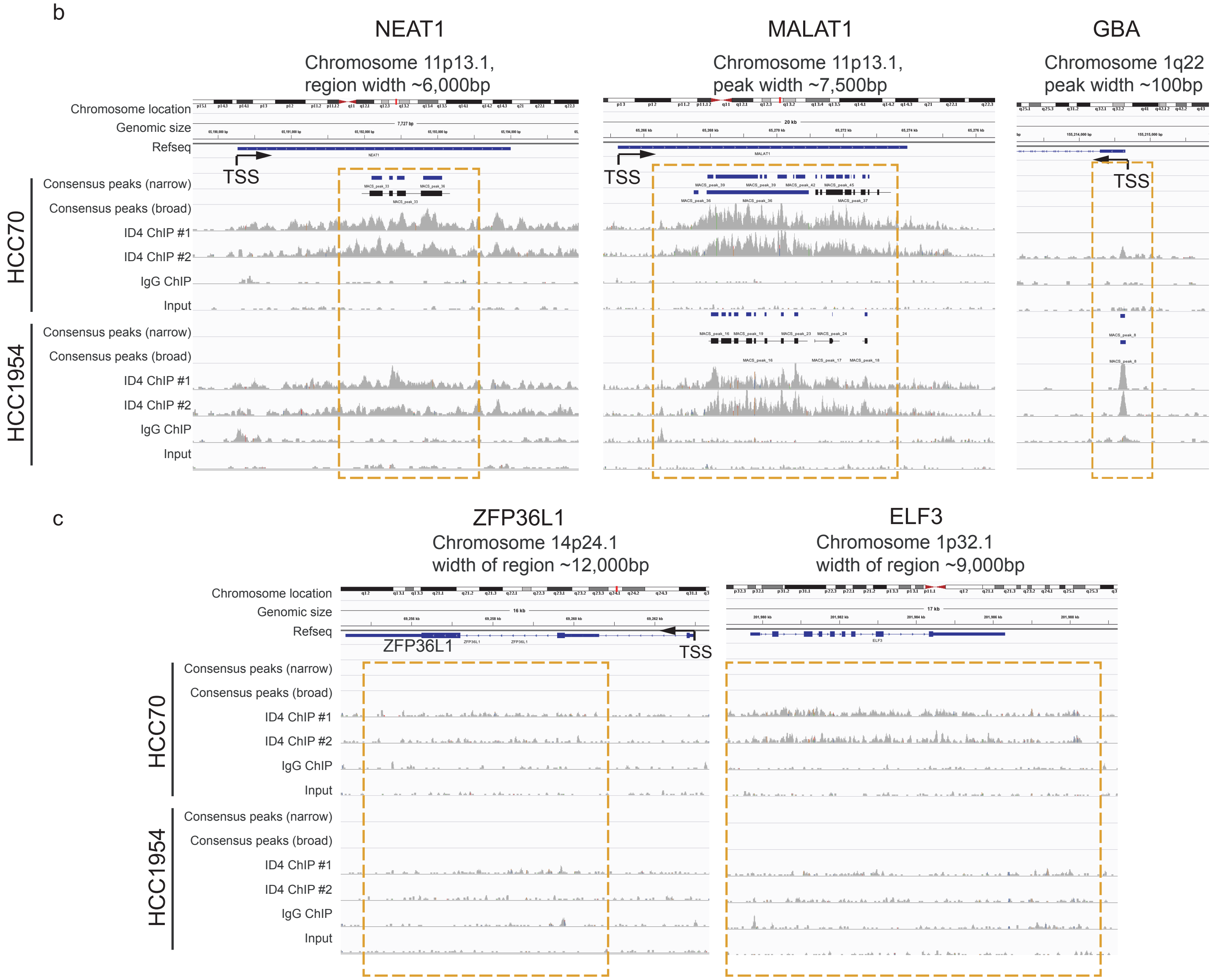

Supplementary Figure 3 cont.

d

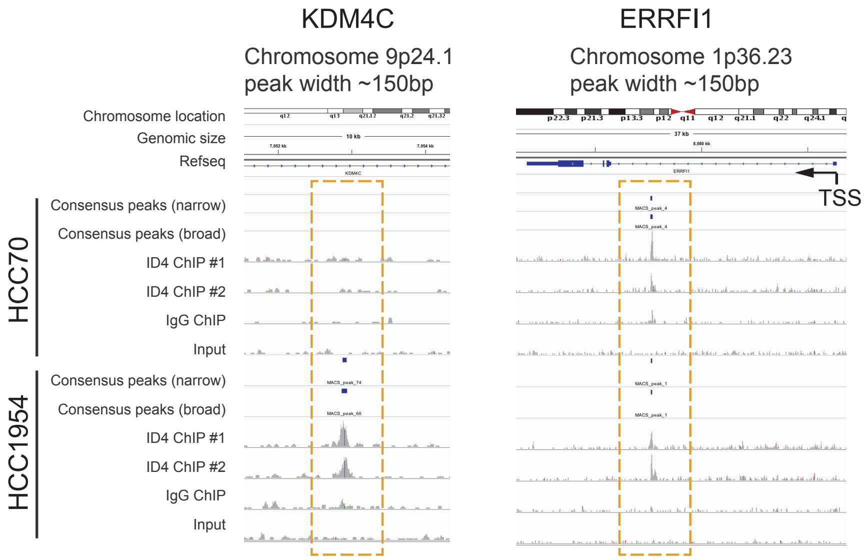

Supplementary Figure 4

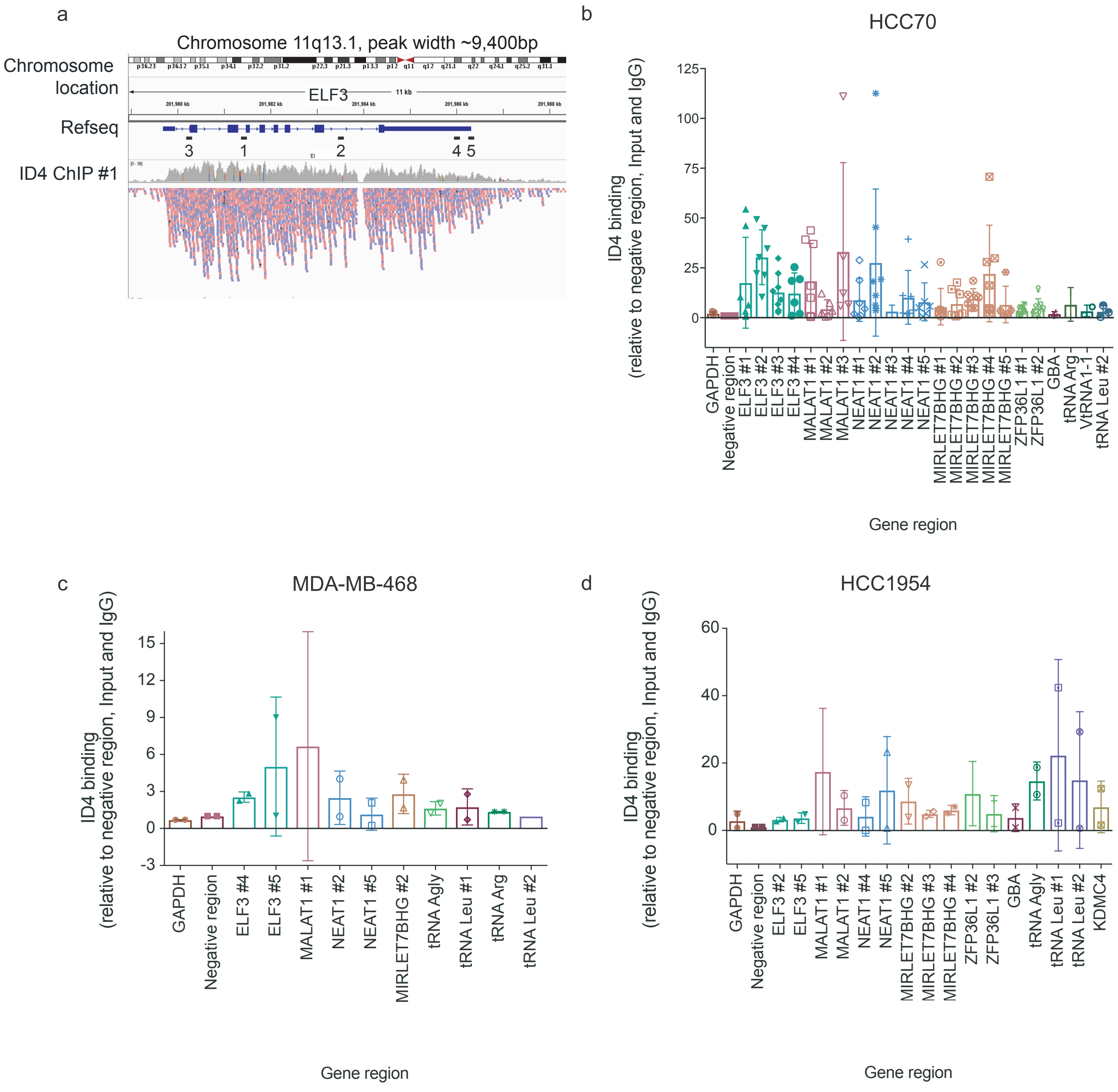

Supplementary Figure 5

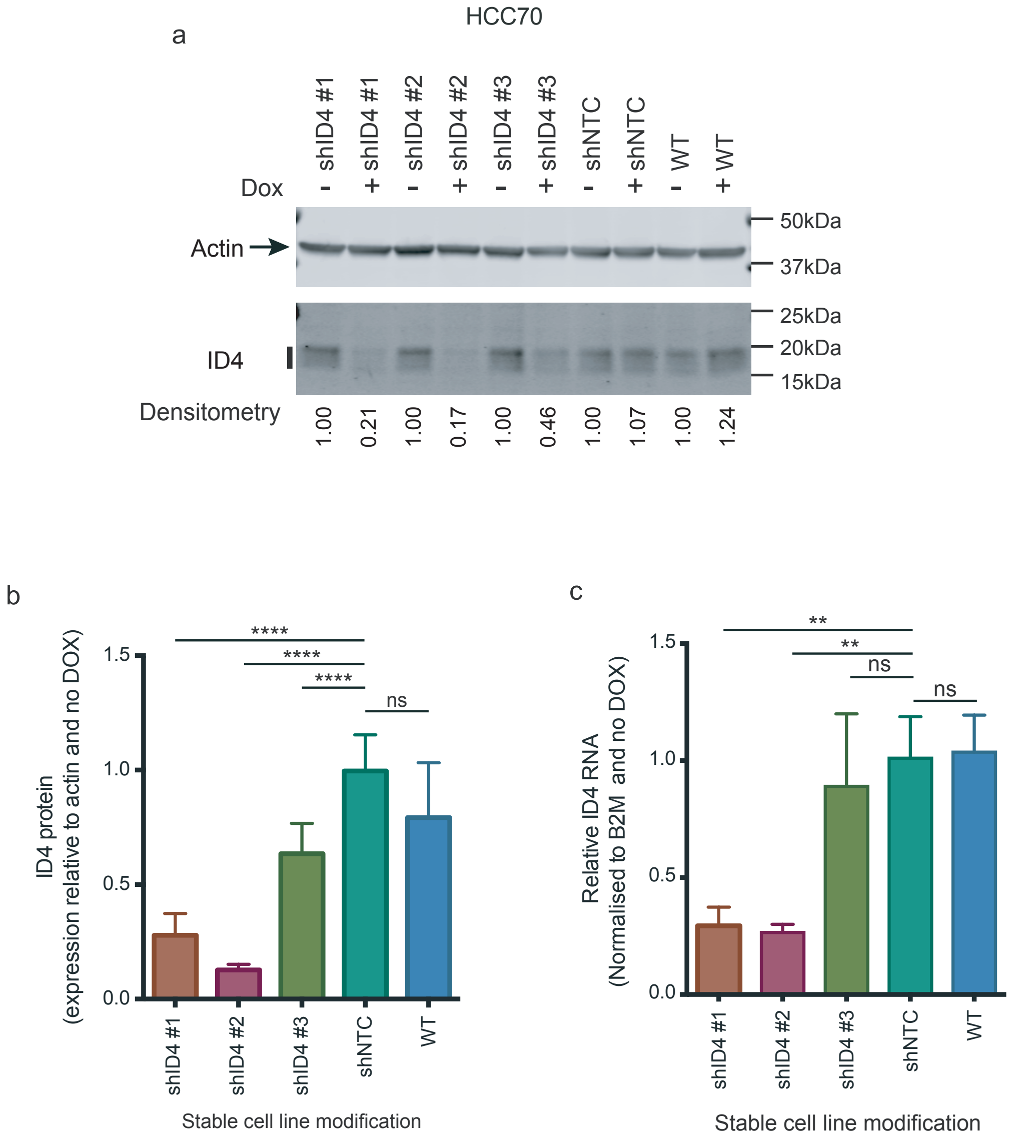

Supplementary Figure 6

a

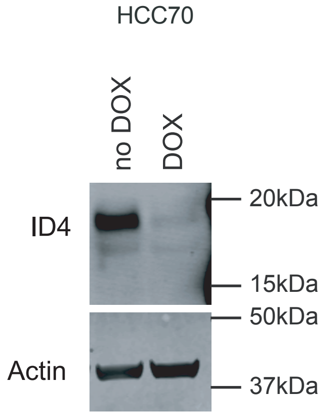

b

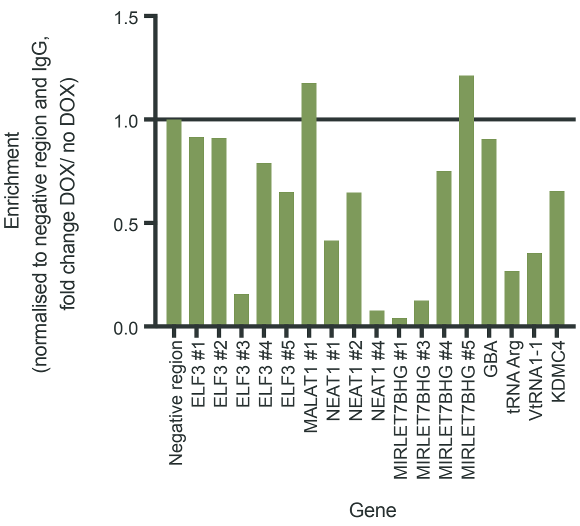

c

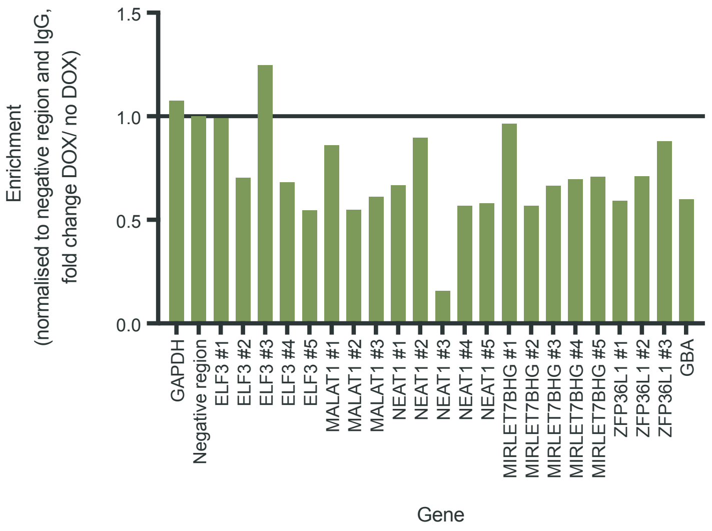

Supplementary Figure 7

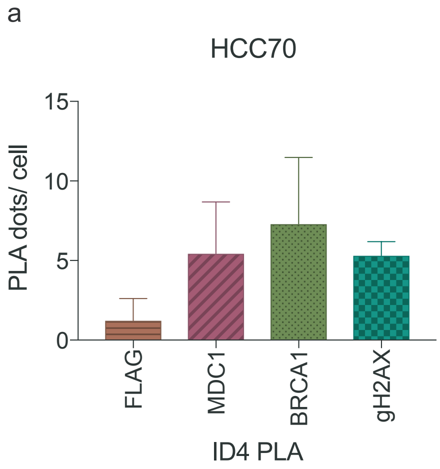

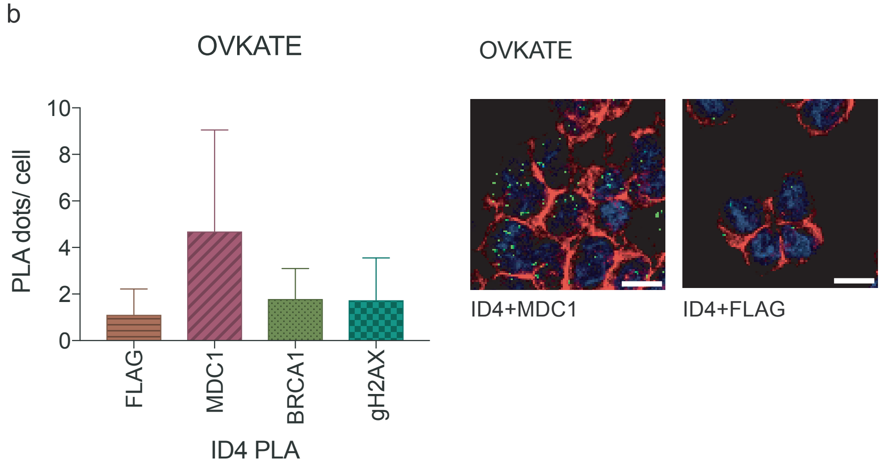

Supplementary Figure 8

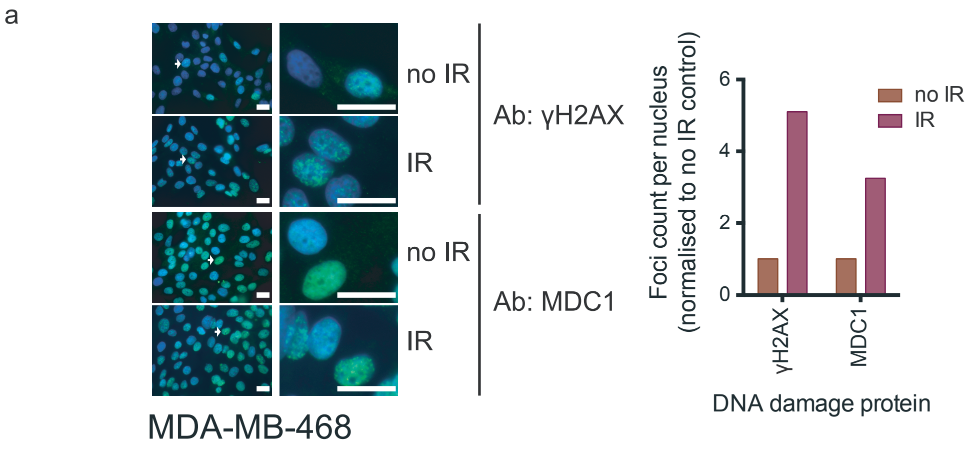

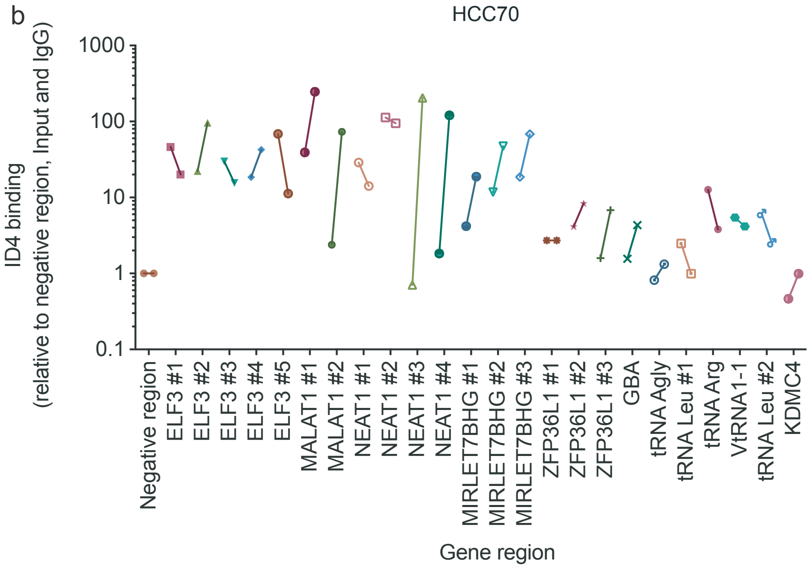

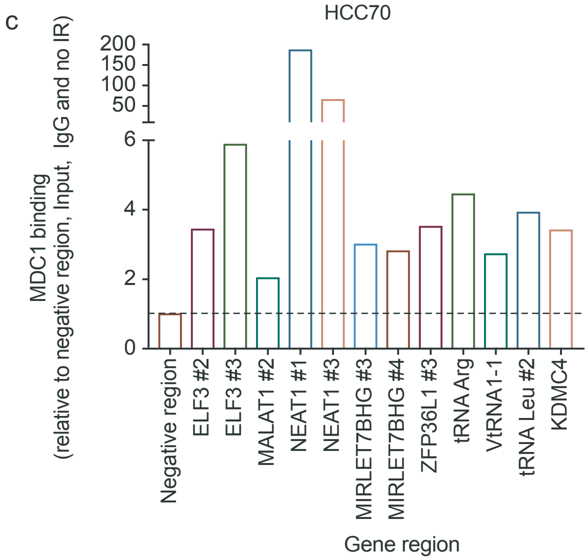

Supplementary Figure 9

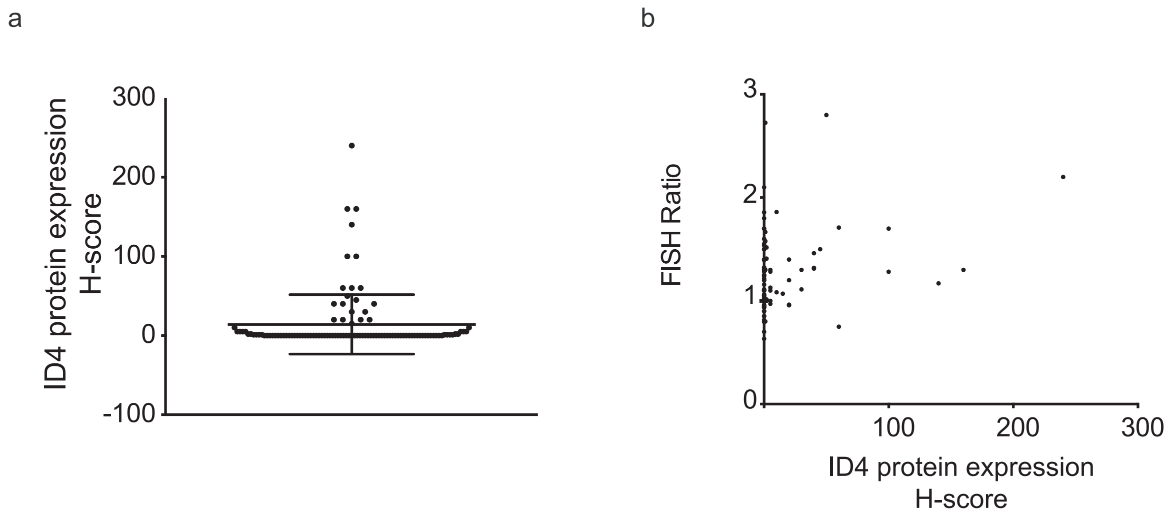

Supplement: Supplementary file 1 — Additional file 1: Figure S1. Analysis of ID4 protein expression across a panel of breast and ovarian cell lines. (A) Western blotting analysis of ID4 protein expression across a panel of breast (top) and ovarian (bottom) normal and cancer cell lines. A rabbit monoclonal antibody specific to ID4 (Biocheck, BCH-9/82–12) was used for detection [11]. Two isoforms of ID4 are detectable across the panel. β-Actin is shown as a loading control. Modifications to images indicated with vertical black line. Cell lines selected for further analysis are indicated with a black square. Breast cancer subtypes and ovarian cancer histotypes indicated [82, 83]. (B) Western blot validation of RIME protocol. MDA-MB-468 cells were processed using the RIME protocol. Antibodies targeting ID4 (pooled polyclonal antibodies) and IgG (rabbit species matched control) control were used for immunoprecipitation. Western blot analysis for ID4 protein expression, using an independent ID4 monoclonal antibody, following immunoprecipitation. Figure S2. ID4 ChIP-sequencing analysis of HCC70 cell line identifies reproducible ID4-chromatin binding sites. (A) Table summarising three biological replicates of ID4 ChIP-seq analysis in HCC70 cell line. (B) 10,000 bp resolution image of ID4 ChIP-seq technical replicate #1 binding to MALAT1 gene. Red sequencing reads are aligned to the positive strand (5′ - 3′), and blue to the negative strand of DNA (3′ - 5′). ID4 binding to (C) left to right: GBA, FAIM, MIRLET7BHG, NEAT1 and ZFP36L1. The chromosomal location, size of the gene and Refseq, human reference genome, are displayed at the top of the image. Reads have been aligned to the human reference genome Hg19 and peaks called using MACs peak calling algorithm (v2.0.9) [38]. Images contain ChIP-seq coverage data and the peaks called for each ID4 technical replicate and the consensus peaks called for all three ID4 ChIP-seq biological replicate for selected gene regions. ID4 binding is shown in comparison to IgG an [file 13058_2020_1306_MOESM1_ESM.pdf]
